# Citrate cross-feeding by *Pseudomonas aeruginosa* supports *lasR* mutant fitness

**DOI:** 10.1128/mbio.01278-23

**Published:** 2024-01-23

**Authors:** Dallas L. Mould, Carson E. Finger, Amy Conaway, Nico Botelho, Stacie E. Stuut, Deborah A. Hogan

**Affiliations:** 1 Department of Microbiology and Immunology, Geisel School of Medicine at Dartmouth, Hanover, New Hampshire, USA; University of Washington, Seattle, Washington, USA

**Keywords:** quorum sensing, citrate, catabolite repression, cross-feeding, LasR, CbrA, CbrB, RhlR, Crc

## Abstract

Cross-feeding of metabolites between subpopulations can affect cell phenotypes and population-level behaviors. In chronic *Pseudomonas aeruginosa* lung infections, subpopulations with loss-of-function (LOF) mutations in the *lasR* gene are common. LasR, a transcription factor often described for its role in virulence factor expression, also impacts metabolism, which, in turn, affects interactions between LasR+ and LasR− genotypes. Prior transcriptomic analyses suggested that citrate, a metabolite secreted by many cell types, induces virulence factor production when both genotypes are together. An unbiased analysis of the intracellular metabolome revealed broad differences including higher levels of citrate in *lasR* LOF mutants. Citrate consumption by LasR− strains required the CbrAB two-component system, which relieves carbon catabolite repression and is elevated in *lasR* LOF mutants. Within mixed communities, the citrate-responsive two-component system TctED and its gene targets OpdH (porin) and TctABC (citrate transporter) that are predicted to be under catabolite repression control were induced and required for enhanced RhlR/I-dependent signaling, pyocyanin production, and fitness of LasR− strains. Citrate uptake by LasR− strains markedly increased pyocyanin production in co-culture with *Staphylococcus aureus*, which also secretes citrate and frequently co-infects with *P. aeruginosa*. This citrate-induced restoration of virulence factor production by LasR− strains in communities with diverse species or genotypes may offer an explanation for the contrast observed between the markedly deficient virulence factor production of LasR− strains in monocultures and their association with the most severe forms of cystic fibrosis lung infections. These studies highlight the impact of secreted metabolites in mixed microbial communities.

**IMPORTANCE:**

Cross-feeding of metabolites can change community composition, structure, and function. Here, we unravel a cross-feeding mechanism between frequently co-observed isolate genotypes in chronic *Pseudomonas aeruginosa* lung infections. We illustrate an example of how clonally derived diversity in a microbial communication system enables intra- and inter-species cross-feeding. Citrate, a metabolite released by many cells including *P. aeruginosa* and *Staphylococcus aureus*, was differentially consumed between genotypes. Since these two pathogens frequently co-occur in the most severe cystic fibrosis lung infections, the cross-feeding-induced virulence factor expression and fitness described here between diverse genotypes exemplify how co-occurrence can facilitate the development of worse disease outcomes.

## INTRODUCTION

In populations formed by diverse cell types, from microbes to human cells, pre-existing variation or *de novo* mutation and selection can lead to subpopulations with different phenotypes (1–3). One important example of co-existing sub-populations is found in chronic *Pseudomonas aeruginosa* infections. Across many distinct *P. aeruginosa* infections, co-occurring subpopulations are differentiated by mutations in common genes or pathways (4–10). Among these common distinctions, loss-of-function mutations in the *lasR* gene are one of the most frequently observed *P. aeruginosa* genotypes (11). LasR is a key part of a microbial communication system referred to as quorum sensing (QS), which impacts diverse phenotypes through an interconnected network of transcription factors including RhlR and PqsR (12). While the *lasR* gene frequently contains loss-of-function mutations, genes encoding other *P. aeruginosa* QS regulators are generally preserved (13). Interestingly, *lasR* mutants are frequently observed amid strains with functional LasR (LasR+) (6, 11, 14). Across diverse species, QS regulation is known to control the production of secreted virulence factors (15). While *lasR* loss-of-function mutants produce lower levels of many virulence factors in monoculture and are less virulent in infection models (16–19), LasR− strains often show increased virulence factor production when interacting with neighboring wild-type (WT) *P. aeruginosa* cells or in co-culture with other species (20, 21). The different interactions that LasR− strains have with other microbes in addition to differences in interactions with host cells (22, 23) may explain why LasR− strains are associated with worse health outcomes (6).

Metabolism is an important factor governing the virulence of many microbes. QS control over metabolism is increasingly recognized (24–26). In fact, several studies support the model that *lasR* loss-of-function mutations confer metabolic advantages that promote fitness (27–29). Our prior experimental evolution studies indicate that the rapid rise of LasR− strains in passaged populations requires the activity of a metabolic regulatory process known as catabolite repression, and that other mutations that lead to reduced catabolite repression are sufficient for enhanced fitness (29). In *P. aeruginosa*, catabolite repression controls substrate utilization through a post-transcriptional process wherein the co-repressor Crc blocks the translation of transcripts for alternative substrate utilization in specific conditions (30, 31). Activity of the CbrAB two-component system relieves Crc-dependent translational repression (32). Thus, the increased CbrAB activity in LasR− strains enables enhanced consumption of several diverse carbon sources, including the metabolites enriched in infections that are the result of production by the host or other microbes (27, 29).

Citrate is among the collection of metabolites secreted by diverse cell types including human cells, yeast, and bacteria, which is better utilized by LasR− *P. aeruginosa* strains (29). Citrate is released by many microbes as part of iron homeostasis or due to imbalances in intracellular metabolites. The presence of several mechanisms for citrate transport in *P. aeruginosa* suggests that citrate is an important metabolite in *P. aeruginosa* biology (33). Citrate was proposed to be a cross-fed metabolite from LasR+ to LasR− cells based on transcriptome changes (20) and is produced by *Staphylococcus aureus* in single species cultures and in co-cultures with *P. aeruginosa* (34). *P. aeruginosa* and *S. aureus* are frequently found together in chronic cystic fibrosis lung infections and are associated with the most severe forms of the disease (35–38).

This paper seeks to specifically examine the role of citrate in intra- and interspecies interactions. The studies presented here highlight the differences between citrate consumption and intracellular accumulation in LasR+ and LasR− strains. We show that citrate uptake relies on the TctABC transporter and the OpdH porin and that these transporters mediate the LasR− strain response to LasR+ strains. The citrate uptake in LasR− strains results from the higher activity of the CbrA-CbrB pathway and reduced Crc-mediated repression of metabolism. Citrate uptake leads to the activation of RhlR-RhlI controlled QS signaling and induction of phenazines and other RhlR-regulated virulence factors, which can antagonize the growth of other cell types and RhlR-I dependent fitness in passaged cultures. The ability of *P. aeruginosa* to take up citrate was a necessary element in interactions with *Staphylococcus aureus*. Together, results from these studies make a case that citrate is an important cross-fed metabolite in microbial populations and communities that shapes *P. aeruginosa* behavior.

## RESULTS

### LasR has broad impacts on the metabolome including effects on intracellular and extracellular citrate concentrations

**Fig 1:**
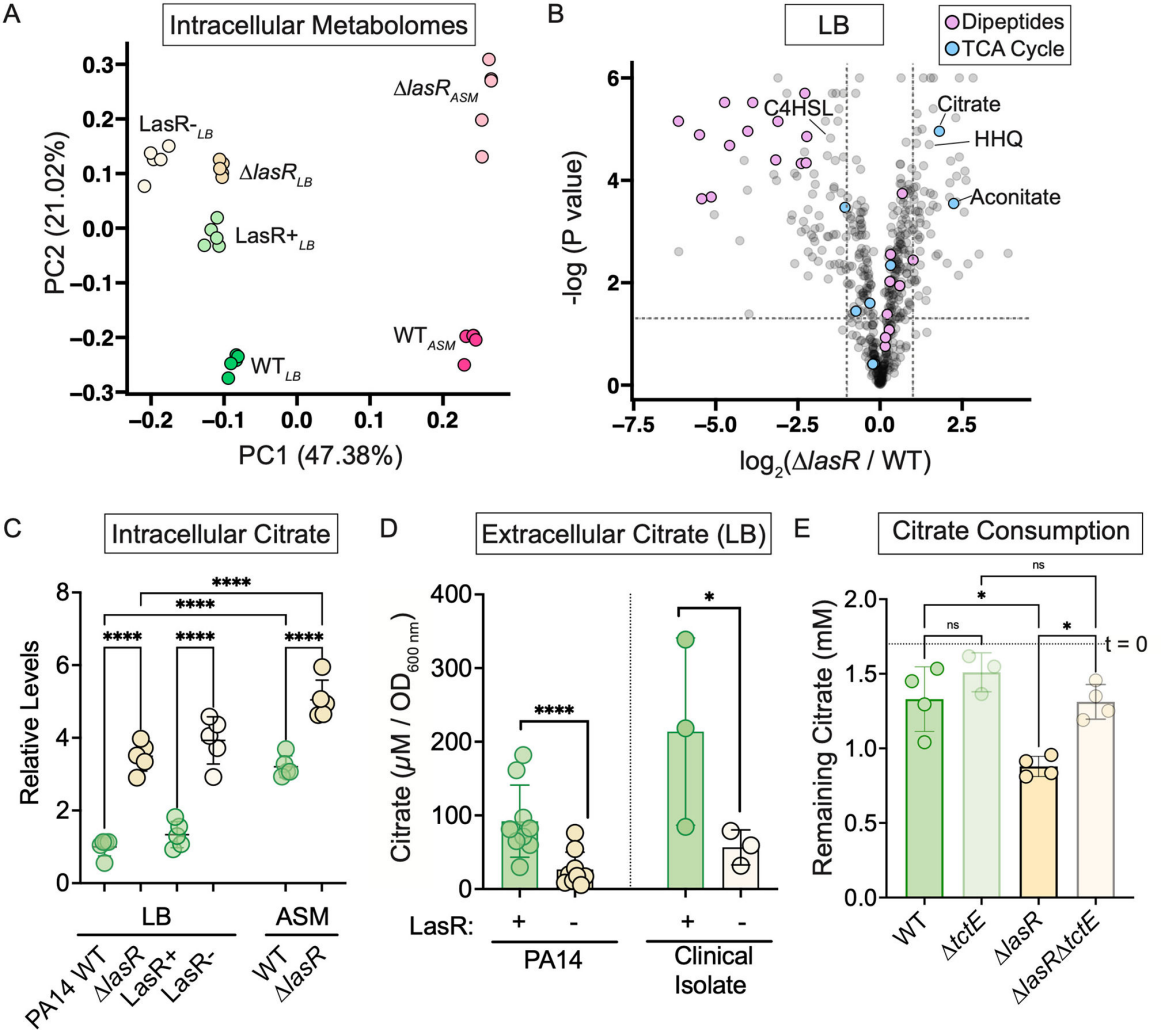
LasR function impacts the metabolic state of the cell promoting differences in citrate homeostasis. (A) Principal component analysis of Pareto-normalized metabolite counts as determined by UPLC-MS/MS of laboratory strains PA14 and Δ*lasR*, and closely related CF clinical isolates with functional LasR (LasR+, DH2417) and dysfunctional LasR (LasR−, DH2415) grown as colony biofilms for 16 h on LB and/or ASM with medium indicated as a subscript (*n* = 5). (B) Volcano plot showing differential metabolite counts (log_2_*lasR*/WT) for Δ*lasR* cells relative to wild-type cells in LB on the *x*-axis; the *y-*axis shows the −log_10_ (*P* value) for the difference between sample types. Metabolites categorized as dipeptides (lavender) or as part of the tricarboxylic acid (TCA) cycle (light blue) are indicated alongside 4-hydroxy-2-heptylquinoline (HHQ) and N-butanoyl-L-homoserine lactone (C4HSL), which are known to be enriched and depleted in *lasR* mutants, respectively. (C) Relative metabolite counts of citrate as determined from the experiment described in panel A for the laboratory strain on LB and ASM and the clinical isolate pair on LB. Statistical significance was determined by ordinary one-way ANOVA with Šidák’s multiple comparisons test: ****P value < 0.0001. (D) Extracellular citrate measured in supernatant of PA14 (*n* = 9), Δ*lasR* (*n* = 9), DH2417 (LasR+) (*n* = 3), and DH2415 (LasR−) (*n* = 3) of 16 h grown cultures in 5 mL of LB using an enzymatic commercial assay. Statistical significance was determined by Student’s *t*-test, ****P value < 0.0001 and *P value < 0.05. (E) Citrate remaining in supernatants taken from densely inoculated cultures of wild type, the Δ*lasR* strain, and derivatives lacking the citrate-responsive sensor kinase TctE incubated for 24 h in LB supplemented with 2 mM citrate as measured via enzymatic assay (*n* > 3). Dotted line indicates citrate concentration recovered at the start of the experiment (*t* = 0). Data included in this graph are a subset of the experiments including data in Fig. 2C. Statistical significance was determined via ordinary one-way ANOVA with Šidák’s multiple comparisons test for entire experimental data sets including all strains: **P value < 0.005; ns, not significant (*P* value = 0.75 and 0.66 for WT vs Δ*tctE* and Δ*tctE* vs Δ*lasR*Δ*tctE* comparisons, respectively).

Toward testing the hypothesis that citrate is a cross-fed metabolite between LasR+ and LasR− cells (20), we first investigated the impact of LasR on *P. aeruginosa* metabolism broadly. To do so, we characterized the intracellular metabolite profiles for colony biofilms of *P. aeruginosa* strain PA14 wild type and its Δ*lasR* derivative, and a pair of clinical isolates that differed by LasR function (DH2417 LasR+ and DH2415 LasR−). Each strain was grown in quintuplicate on lysogeny broth (LB). Analysis of the intracellular metabolomes, performed by ultrahigh performance liquid chromatography-tandem mass spectroscopy (UPLC-MS/MS), revealed broad differences between LasR+ and LasR− cells. Of the 562 detected metabolites with known identities, over 100 were significantly differentially abundant in each comparison: Δ*lasR*/WT on LB and DH2415 (LasR−)/DH2417 (LasR+) on LB (Table S1). We extended this analysis by looking at the PA14 and Δ*lasR* strains on artificial sputum medium (ASM) (39, 40), which mimics the cystic fibrosis (CF) lung environment where *lasR* loss-of-function mutants frequently emerge (data in Table S1). In a principal component analysis of the normalized metabolite counts, the first component, which explained 47.4% of the variation in the data, separated samples by medium type, while the second component, explaining 21% of the variation in the data, appeared to separate samples by LasR functional status (Fig. 1A). The clinical isolates clustered more closely to the Δ*lasR* strain than the wild type on LB but still showed separation by LasR type along the second principal component axis (Fig. 1A).

**Fig 2:**
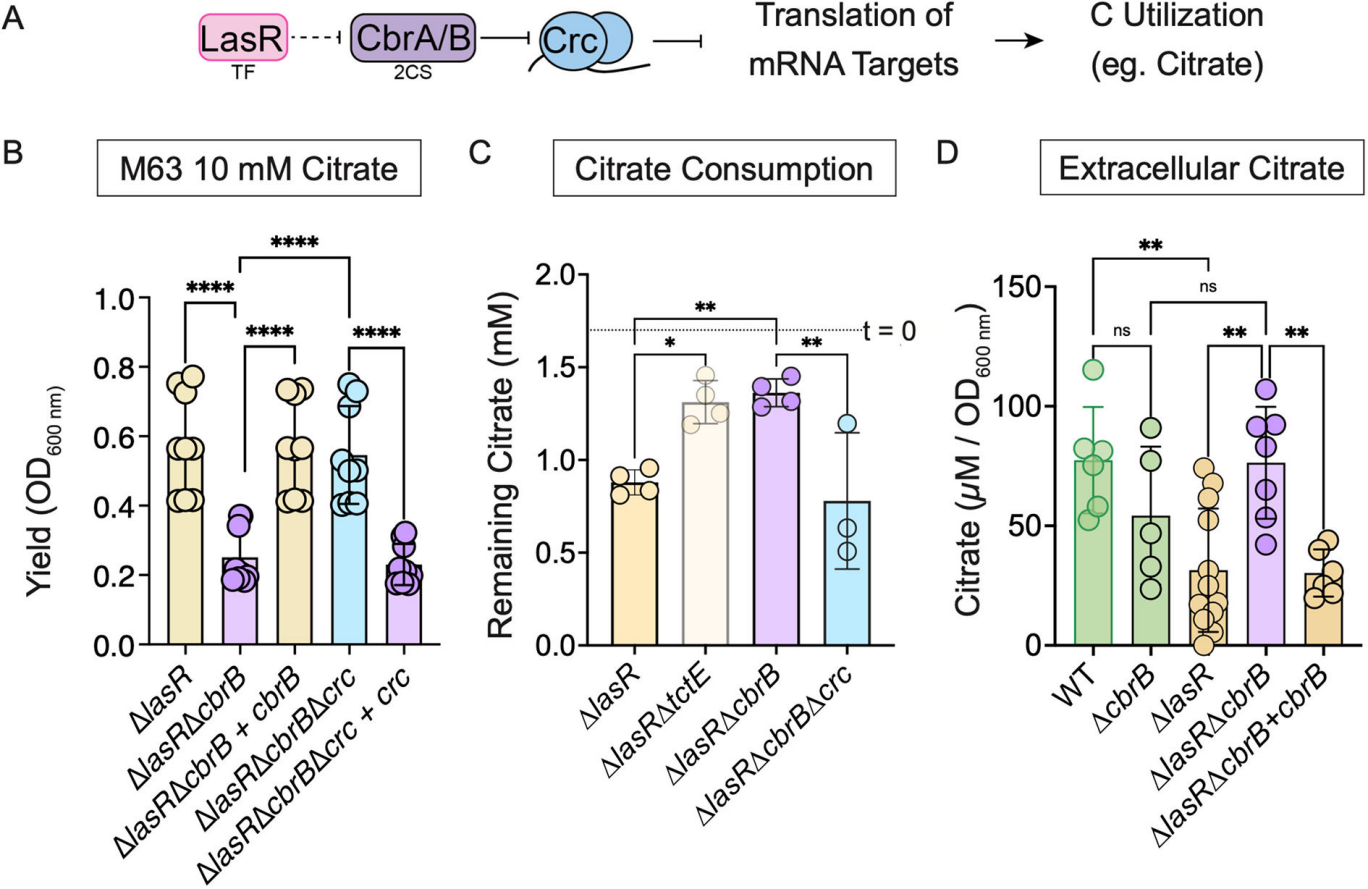
Catabolite repression controls citrate consumption but not citrate secretion enabling preferential uptake by LasR− cells. (A) Simplified schematic illustrating the connection between the transcription factor LasR and the carbon catabolite repression pathway, including the two-component system CbrAB, which promotes de-repression of mRNA targets important for alternative carbon (C) utilization such as citrate for which genes involved in its consumption are predicted to be under Crc translational inhibition. (B) Growth of indicated strains on 10 mM citrate in M63 base after 16 h following subculture from LB overnight. Data points indicate three biological replicates of three independent experiments. Statistical significance as determined by ordinary one-way ANOVA with Šidák’s multiple comparisons test: ****P value < 0.0001. (C) Citrate consumption after 24-h incubation in LB supplemented with 2 mM citrate for the Δ*lasR* strain and derivatives lacking the sensor kinase TctE, the response regulator CbrB, and/or the translational co-repressor Crc of the catabolite repression system. Each data point is from an independent experiment (*n* > 3). Dotted line indicates citrate concentration recovered at the start of the experiment (*t* = 0). Data included in this graph are a subset of the experiment including data in Fig. 1E. Statistical significance was determined via ordinary one-way ANOVA with Šidák’s multiple comparisons test including all data from the experiment: *P value = 0.02 and **P value < 0.01. (D) Extracellular citrate levels (µM/OD_600 nm_) in supernatants from the indicated strains grown in LB as measured via an enzymatic assay. Statistical significance as determined by ordinary one-way ANOVA with Šidák’s multiple comparisons test: **P value < 0.005 and ns, not significant with *P* value > 0.44 (*n* > 5).

The metabolomics data were consistent with the well-described roles for LasR in the control over the PqsR- (MvfR-) and RhlR-dependent regulons of the inter-connected quorum-sensing system and the positive regulation of protease production. For example, LasR regulates the enzyme PqsH that converts 4-hydroxy-2-heptylquinoline (HHQ) into 2-heptyl-3-hydroxy-4(1H)-quinolone, and loss of *lasR* leads to the accumulation of HHQ, which gives *lasR* mutant colonies their characteristic sheen or metallic appearance (27). We found that HHQ was significantly more abundant in LasR− cells when compared to the wild type for the PA14 background on LB (Fig. 1B) and ASM (Fig. S1A), and to a lesser extent with the clinical isolate background on LB (Fig. S1B). Also, consistent with LasR-dependent production of the autoinducer N-butanoyl-L-homoserine lactone (C4HSL) synthase RhlI (regulated by RhlR), we found significantly lower levels of C4HSL in LasR− cells relative to wild type on LB (Fig. 1B; Fig. S1B). Of note, unlike the LB samples, C4HSL was not significantly differentially abundant between strains on ASM at this time point (Fig. S1A). Lastly, over 50% of all dipeptides detected was significantly less abundant in *lasR* mutant cells consistent with the lower proteolytic activity present in LasR− strains (41, 42) (Fig. 1B). Similar observations were made when comparing the wild type and Δ*lasR* strain on ASM and the LasR+ and LasR− clinical isolates on LB (Fig. S1A and B).

The broad differences in the metabolite profiles between LasR+ and LasR− cells suggest different anabolic and catabolic states when grown on the same medium type, which creates the potential for cross-feeding or differential substrate utilization between strains. Tricarboxylic acid (TCA) cycle intermediates, including citrate, are critical for both energy generation and biosynthesis and are frequently secreted by microbes and host cells under particular conditions like iron or oxygen limitation (43). While there is potential for several metabolites to be cross-fed, we focused our efforts on citrate with the goal of understanding if it is managed differently in LasR+ and LasR− cells given our prior work that found that citrate supplementation induces RhlR-dependent transcriptional activity in LasR− strains but not LasR+ strains (20). We found that in both strain backgrounds on LB and in strain PA14 on ASM, citrate stood out among the TCA intermediates with a fold change > 1.6 and *P* value < 0.05 in all conditions (Fig. S1C). Succinate was also significantly different in all conditions, but the fold differences were much smaller (Fig. S1C). Intracellular citrate was significantly more abundant in LasR− cells than those with intact LasR on LB and ASM (Fig. 1C).

To complement the analysis of intracellular citrate, we measured extracellular citrate levels in cell-free supernatants from the same sets of LasR+ and LasR− isolates as above after 16 h of growth in the LB medium. In fresh, uninoculated LB medium, we detected 100 ± 103 µM (average ± s.d.) citrate (Fig. S2). After growth, PA14 wild-type culture supernatants contained 327 ± 89 µM citrate, while supernatants of Δ*lasR* cultures contained only 87 ± 58 µM citrate (Fig. S2). The significantly higher levels of citrate in supernatants of wild-type cultures than in the LB medium blank suggested that wild-type cells secreted citrate. In contrast, there was no significant difference in citrate levels between the LB medium and supernatant from Δ*lasR* cultures (Fig. S2). The differences in citrate between strain supernatants were not due to the differences in growth as optical density at 600 nm (OD_600_)-normalized citrate levels were significantly lower (by three- to fourfold) in the supernatants of LasR− strains when compared to those for their respective LasR+ strains (Fig. 1D). Overall, relative to LasR+ cells, LasR− cells had higher intracellular citrate levels and less extracellular citrate, suggesting LasR function may impact citrate consumption and/or secretion.

To monitor citrate consumption, we added 2 mM citrate to high-density cultures in LB medium and monitored the amount of citrate remaining after 24 h. As a control, we included strains lacking the gene encoding the histidine kinase TctE, which regulates genes involved in citrate uptake as part of a two-component system (see Fig. S3A for pathway) (33, 44, 45). In the supernatant of the Δ*lasR* strain, there was significantly less citrate recovered than for the wild type (Fig. 1E). The depletion of citrate by LasR− strains required *tctE*, and there was no significant difference between the Δ*tctE* and Δ*lasR*Δ*tctE* strains (Fig. 1E). Furthermore, there was no significant difference in the amount of citrate remaining in the supernatant of PA14 wild type compared to supernatant from the Δ*tctE* strain suggesting minimal net citrate consumption by the wild type (Fig. 1E). Collectively, the data suggest differential consumption of citrate by LasR+ and LasR− strains.

### Reduced catabolite repression via CbrB-Crc control promotes TctED-dependent citrate consumption by LasR− cells

LasR− strains have increased activity of the CbrAB two-component system that enables de-repression of the catabolism of diverse substrates including citrate by modulating translational inhibition by Crc (27, 29) (see Fig. 2A for pathway). Genetic analyses demonstrate the control of citrate catabolism by CbrAB and Crc in the Δ*lasR* mutant (Fig. 2B). Growth of the Δ*lasR* strain on citrate was significantly reduced in the absence of *cbrB*, and this could be complemented by restoration of *cbrB* at the native locus. Subsequent deletion of *crc* in the Δ*lasR*Δ*cbrB* strain also rescued growth on citrate, and this phenotype was reversed by complementation of *crc* (Fig. 2B). The CbrB-Crc control of growth on citrate mirrored the effects by CbrB and Crc on citrate uptake by Δ*lasR* cells in LB (Fig. 2C). The Δ*lasR* mutant depleted more citrate than did the Δ*lasR*Δ*cbrB* strain, and again the Δ*lasR*Δ*cbrB* phenotype was relieved by the deletion of *crc* (Fig. 2C). The Δ*lasR*Δ*cbrB* defect in citrate depletion was similar to that observed for the Δ*lasR*Δ*tctE* strain (Fig. 2C). Multiple lines of evidence indicate that the TctE-regulated genes *opdH-tctA-tctB-tctC* important for citrate utilization are under Crc control: (i) there is a putative *crc* binding site motif upstream of the translational start site (46); (ii) transcript levels of genes in this operon are elevated in *crc* mutants (44, 46); (iii) OpdH levels are elevated in strains lacking *crc* (47); and (iv) paired transcriptome and proteome analyses predict *tctC* and *opdH* as Crc targets (48).

Given the requirement for CbrB in selective citrate consumption, we attempted to assess its impact on net secretion to obtain a clearer grasp on citrate management by each cell type. We measured extracellular citrate in strains lacking *cbrB*, which was shown to impede uptake (Fig. 2C). While the wild type had significantly more citrate than the Δ*lasR* strain, the levels of citrate were relatively high in both backgrounds when *cbrB* had been deleted (Fig. 2D). The significant increase in citrate levels observed in the Δ*lasR*Δ*cbrB* strain relative to the Δ*lasR* strain dropped to the Δ*lasR* strain levels when *cbrB* was complemented at the native locus (Fig. 2D). The loss of *cbrB* in the wild type, on the other hand, did not significantly impact extracellular citrate levels, though the data may suggest that CbrB does play some condition-specific role in citrate secretion by the LasR+ strain (Fig. 2D).

### In co-culture, tricarboxylates are detected by the TctED two-component system of LasR− strains

**Fig 3:**
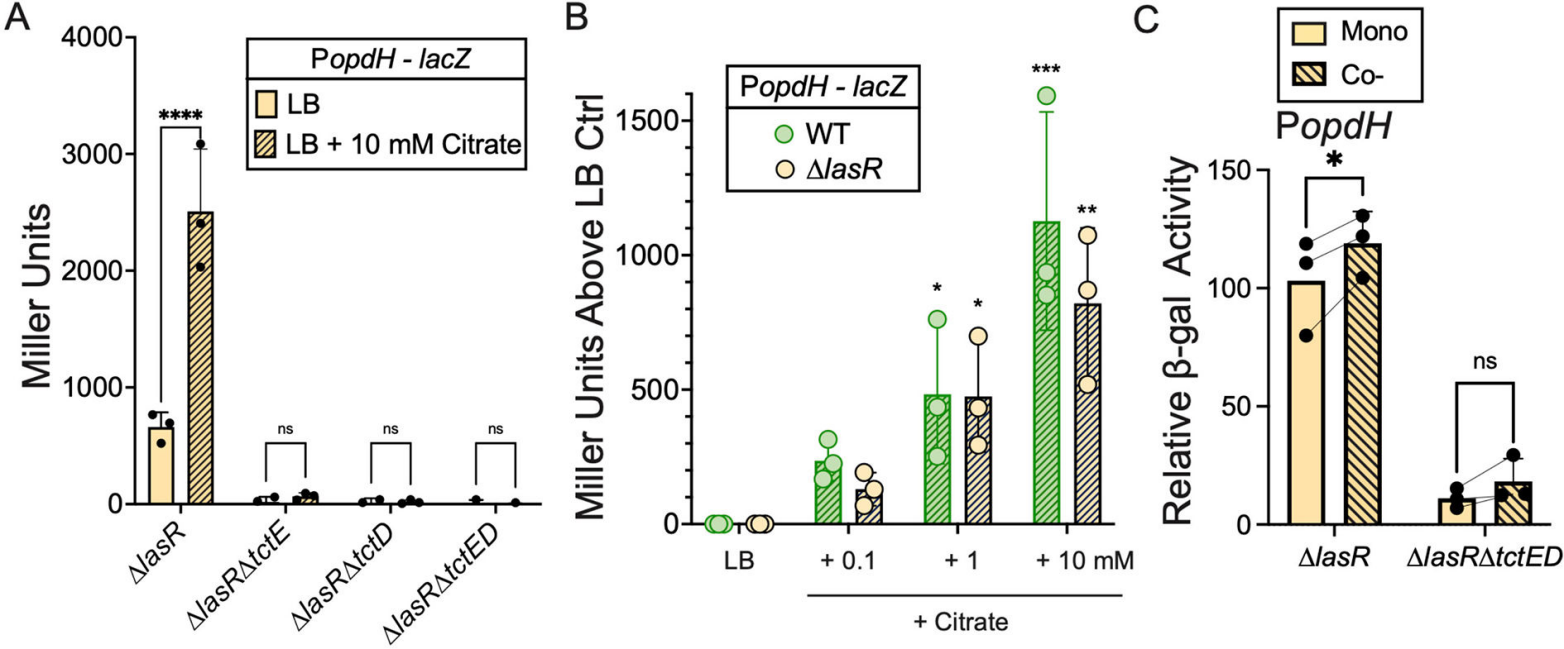
Citrate is detected in co-cultures of LasR+ and LasR− strains. (A) Citrate-responsive P*opdH–lacZ* activity quantified by beta-galactosidase (β-gal) assay of Δ*lasR*, Δ*lasR*Δ*tctE*, Δ*lasR*Δ*tctD*, and Δ*lasR*Δ*tctED* colony biofilms on LB or LB supplemented with 10 mM citrate. Statistical significance as determined by two-way ANOVA with Šidák’s multiple comparisons test: ****P value < 0.0001 and ns, not significant (*P* value > 0.99). (B) Promoter activity quantified for P*opdH*–GFP–*lacZ* of WT and Δ*lasR* colony biofilms grown on LB supplemented with 100 µM, 1 or 10 mM of citrate after 16 h of growth (*n* = 3) via β-gal assay. Statistical significance was determined by two-way ANOVA (matching for citrate concentration and replicate) with Šidák’s multiple comparisons test. There was no significant source of variation detected between strains (*P* value = 0.53), but there were differences noted across citrate concentrations (*P* value = 0.0004). Asterisks denote statistical significance for comparison to LB control: *P value < 0.04; **P value < 0.003; and ***P value = 0.0005. (C) β-gal quantification of P*opdH*–GFP–*lacZ* in Δ*lasR* and Δ*lasR*Δ*tctED* strains in monoculture or co-culture with fluorescently (mKate) labeled WT. Miller units were adjusted for the proportion of the cellular density containing a strain with a *lacZ* reporter at the time of quantification. Statistical significance as determined by two-way ANOVA with Šidák’s multiple comparison’s test: *P value = 0.0326 and ns, not significant (*P* value = 0.2721).

In light of our data that suggested *lasR* mutants are capable of both producing and consuming citrate in LB medium while the wild-type cells only showed evidence for citrate release, we sought to determine if wild-type and Δ*lasR* strains exhibited similar transcriptional responses to citrate. To do so, we used a transcriptional reporter for the promoter upstream of the *opdH-tctA-tctB-tctC* operon, which is induced in wild type (LasR+ backgrounds) by TctED in response to citrate (33, 44, 45). Consistent with the TctE-dependent growth (Fig. S3B) and consumption of citrate by *lasR* mutants (Fig. 1E), *tctE* and *tctD* were both required to induce the *opdH* promoter in response to citrate in a LasR− strain background (Fig. 3A), much like reports in LasR+ strains (33). Complete induction of *opdH* promoter activity by citrate in *lasR* mutants required citrate entry into the cell, as the absence of the porin OpdH reduced induction by citrate relative to Δ*lasR* strain, and the absence of the inner membrane transporter TctABC rendered the *opdH* promoter completely non-responsive to citrate (Fig. S3C).

To assess the sensitivity of *opdH* promoter induction between strains, we considered citrate concentrations within the range of that observed in supernatant from wild-type (LasR+) monocultures (∼200–500 µM). Citrate-induced P*opdH–lacZ* activity above baseline in both wild-type and Δ*lasR* cells when added to LB at low concentrations (+100 µM) with more significant differences as citrate concentrations increased (Fig. 3B). Furthermore, we found significantly greater TctED-dependent induction of the *opdH* promoter fusion in Δ*lasR* cells when grown in co-culture with the LasR+ cells than when grown alone, suggesting Δ*lasR* cells were responding to the higher concentrations of extracellular citrate released by LasR+ cells (Fig. 3C).

### CbrAB-Crc regulation is required for the induction of RhlR signaling and RhlR-regulated pyocyanin production in LasR− strains grown in co-culture with LasR+ strains

**Fig 4:**
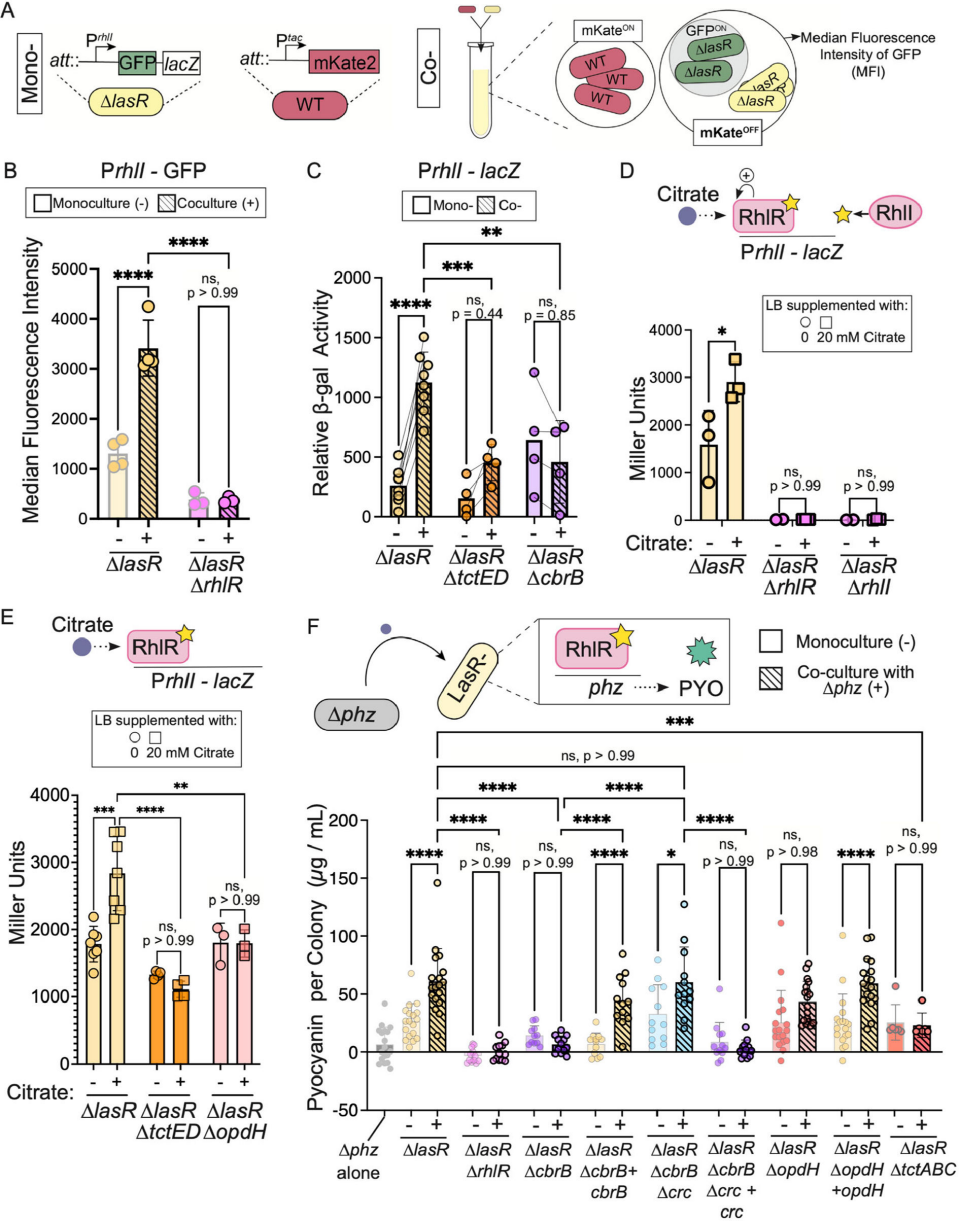
Redundant citrate uptake is required for the activation of RhlR signaling and phenazine production by LasR− strains in co-culture. (A) Graphic illustration of experimental set up to measure *rhlI* promoter activity of LasR− cells via flow cytometry. The Δ*lasR* P*rhlI*–GFP–*lacZ* was grown in LB as monocultures or co-cultures with a wild-type strain expressing mKate2 constitutively under the P*tac* promoter. Median fluorescence intensity (MFI) of GFP (GFP^ON^) was monitored as a readout of *rhlI* promoter activity specific to LasR− cells in co-culture given they lack mKate2 (mKate^OFF^). (B) MFI of P*rhlI*–GFP–*lacZ* for the indicated strains in monoculture (gray outline) or co-culture (black outline) after 6 h in LB as described in panel A. (C) Relative P*rhlI–lacZ* promoter activity quantified by beta-galactosidase assay for the indicated *lasR* mutant derivatives in monoculture or co-culture with mkate2-labeled WT (lacking a *lacZ* reporter). Miller units were adjusted as previously noted. (D) RhlR regulates its cognate autoinducer (star) synthase RhlI in a positive feedback loop. P*rhlI–lacZ* promoter activity quantified for Δ*lasR* strains lacking the regulator RhlR, synthase RhlI, (E) two-component system TctED, or porin OpdH on LB agar (−) and LB agar supplemented with 20 mM citrate. (F) Pyocyanin (PYO), one RhlR/I-regulated product induced in LasR− strains in co-culture, quantified (µg/mL) per colony biofilm for monocultures of a strain lacking both phenazine biosynthesis operons (Δ*phz*, gray), the Δ*lasR* strain, and its mutant derivatives, as indicated, relative to co-cultures (black outline, hashed bars) of the *lasR* mutants grown with the Δ*phz* strain. Data points are from at least three independent experiments with two biological replicates each. Statistical significance was determined via ANOVA with Šidák’s multiple comparisons test. *P* values (*P*) as indicated.

We have previously reported that in LasR+/LasR− co-cultures, the presence of LasR+ cells induces RhlR activity in LasR− cells due to a factor other than acylhomoserine lactone autoinducers (20). Here, we sought to determine if citrate uptake was required for the induction of RhlR-dependent signaling and phenazine production in LasR− strains when cultured with their LasR+ counterparts. We monitored *rhlI* promoter activity in both single-strain and mixed-strain cultures at both cellular- and community-level resolution using a GFP–*lacZ* dual reporter readout in *lasR* mutants. While *lacZ* and beta-galactosidase (β-gal) assays could provide community-level assessments of *rhlI* promoter activity in LasR− strains, green fluorescent protein (GFP) and flow cytometry could provide insight at the cellular level. Specifically, we used flow cytometry to monitor the median fluorescence intensity of GFP for single *lasR* mutant cells in monoculture and co-culture with LasR+ strains engineered to constitutively express the mKate2 fluorophore (see Fig. 4A for the schematic of the experimental strategy). The median GFP fluorescence indicative of RhlI promoter activity in LasR− cells was determined for cells lacking mKate2 (mKate^OFF^) (Fig. 4A). RhlR signaling in the Δ*lasR* strain increased when assessed at the cellular level via flow cytometry (Fig. 4B) and at the community level with the beta-galactosidase assay (Fig. 4C) when grown with “wild type.” TctED and CbrB, which are required for citrate consumption, were also required for co-culture-induced *rhlI* promoter activity in LasR− cells further implicating citrate as an inducer in co-culture (Fig. 4C). In a Δ*lasR*Δ*rhlR* strain, *rhlI* promoter activity was not observed, as expected, in co-culture (Fig. 4B) nor on LB in the absence or presence of citrate (Fig. 4D). Given that RhlR can be induced in the absence of its cognate autoinducer synthase RhlI (30, 49–54), we tested whether RhlI was necessary for citrate-induced, RhlR-dependent transcriptional activity. Consistent with canonical RhlR activation and our prior report illustrating increased RhlI protein in response to citrate (20), we found that RhlI was required for the response to citrate (Fig. 4D). The induction of *rhlI* promoter activity by citrate depended on TctED and one of its gene targets *opdH* implicated in citrate uptake (Fig. 4E). Succinate, another growth-supporting metabolite elevated in LasR− strains, did not promote increases in RhlI promoter activity, suggesting enhanced growth cannot explain RhlR stimulation by citrate (Fig. S4A). Furthermore, loss of RhlR did not impact the growth on citrate, suggesting citrate consumption is upstream of RhlR/I activation (Fig. S4B).

RhlR regulates phenazine production, which has been shown to be induced in LasR− strains grown with LasR+ strains. Thus, we also monitored the production of one clinically discussed phenazine, pyocyanin, by LasR− strains when grown as colony biofilms in monoculture and co-culture with a LasR+ strain lacking both phenazine biosynthesis operons (Δ*phzA1* Δ*phzB1* Δ*phzC1* Δ*phzD1* Δ*phzE1* Δ*phzF1* Δ*phzG1* Δ*phzA2* Δ*phzB2* Δ*phzC2* Δ*phzD2* Δ*phzE2* Δ*phzF2* Δ*phzG2*) referred to as the “Δ*phz*” strain. By co-culturing the Δ*phz* strain with the Δ*lasR* strain and its derivatives, we are able to specifically measure LasR− strain phenazine production even in co-culture. Consistent with prior work (20), when the Δ*lasR* strain was co-cultured with the Δ*phz* strain (i.e., Δ*lasR*, +), there was an approximate fivefold increase in pyocyanin compared to the Δ*lasR* monoculture (i.e., Δ*lasR*, −) (60 versus 14 µg/mL of pyocyanin per colony biofilm) (Fig. 4F). Consistent with RhlR regulation of phenazine biosynthesis (20), no pyocyanin was recovered from the Δ*lasR*Δ*rhlR* monoculture or co-culture with the Δ*phz* strain (Fig. 4F).

To determine if the increased activity of CbrB in LasR− strains was necessary for phenazine production, we grew the Δ*lasR*Δ*cbrB* strain, which exhibits a more repressed metabolism, in monoculture and in co-culture with the Δ*phz* strain. Colony biofilms of the Δ*lasR*Δ*cbrB* monoculture produced pyocyanin at concentrations similar to that for Δ*lasR* monocultures (Fig. 4F). However, unlike the Δ*lasR* strain, pyocyanin production by the Δ*lasR*Δ*cbrB* mutant was not induced in the presence of the Δ*phz* strain and was significantly lower than when the Δ*lasR* strain was co-cultured with the Δ*phz* strain (Fig. 4F). When the *cbrB* gene was complemented in the Δ*lasR*Δ*cbrB* strain (Δ*lasR*Δ*cbrB + cbrB*), co-culture pyocyanin production was restored (Fig. 4F).

Furthermore, when the Δ*lasR*Δ*cbrB* strain was modified to delete *crc*, which encodes a co-repressor of the catabolite repression system that is downstream of CbrAB, co-culture pyocyanin production was also restored (Fig. 4F). While Δ*lasR*Δ*cbrB*Δ*crc* monocultures produced slightly higher pyocyanin levels than monocultures of the Δ*lasR* strain, the levels increased from 29 µg/mL in monoculture to 63 µg/mL of pyocyanin in co-culture with the Δ*phz* strain (Fig. 4F). To ensure the restoration of the co-culture phenazine response was due to the loss of Crc-mediated repression, we complemented *crc* in the Δ*lasR*Δ*cbrB*Δ*crc* strain, and pyocyanin levels resembled that of the Δ*lasR*Δ*cbrB* strain with low to undetectable amounts and no significant difference between mono- and co-culture levels (Fig. 4F). Together, these data indicate that relief from catabolite repression promotes interstrain interactions, which increase the overall pyocyanin concentration produced by the Δ*lasR* strain. We propose that the induction of RhlR signaling and its downstream targets is the result of a metabolite present in co-culture that requires CbrB for uptake or catabolism.

### Citrate secreted by wild type can support growth and RhlR induction of LasR− strains

Citrate is a metabolite that requires CbrB for utilization, is secreted by *P. aeruginosa*, and is differentially taken up from rich medium by LasR+ and LasR− strains. *lasR* mutant derivatives lacking either the gene encoding a porin shown to transport citrate (OpdH) or the TctABC citrate transporter required for growth on citrate (Fig. S3B) did not exhibit significant co-culture phenazine induction (Fig. 4F), and the defect in co-culture phenazine production by the Δ*lasR*Δ*opdH* strain was rescued by *opdH* complementation (Fig. 4F). The resulting shift in pyocyanin production in co-culture in the absence of CbrB or the genes involved in citrate uptake could be the result of differences in quorum-sensing activity and/or shifts in growth and competitive fitness.

**Fig 5:**
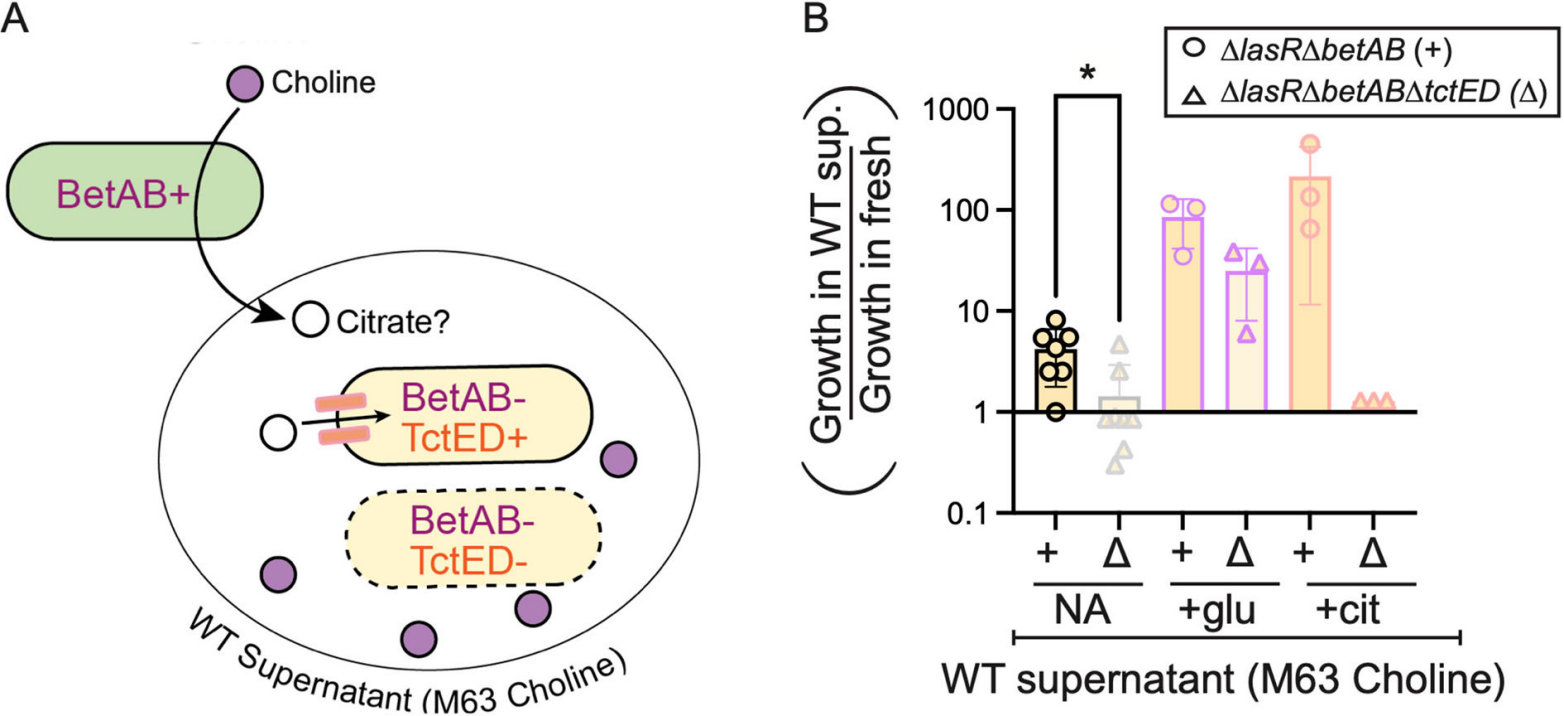
Wild-type-secreted factors support *tctED*-dependent growth of *lasR* mutants on an inaccessible carbon source abundant in airway surfactant. (A) Schematic of the experimental set up. Choline (purple circle) catabolism requires the enzymes BetA and BetB. Strains lacking these enzymes (BetAB−) do not grow well on choline as a sole carbon source but can consume products released by BetAB+ LasR+ strains (green oval). Citrate (open circle), one potential secreted metabolite, is taken up by LasR− BetAB− cells (beige oval) in a TctED-dependent manner. (B) Growth of the Δ*lasR*Δ*betAB* strain (circle, +) or Δ*lasR*Δ*betAB*Δ*tctED* strain (triangles, Δ) lacking the citrate-responsive two-component system controlling citrate uptake in supernatant from wild-type (BetAB+) cultures on M63 medium with choline as a sole carbon source diluted 1:1 in fresh choline medium without additional carbon supplements (NA) or with additional glucose (“+ glu”) or citrate (“+ cit”) relative to fresh 10 mM choline medium. Significance as determined by mixed-effects model with Šidák’s multiple comparisons test: *P < 0.05.

To directly explore the possibility that Δ*lasR* mutants were able to use *P. aeruginosa*-secreted citrate to support growth, we designed an experiment where only the wild type can grow on the provided growth substrate, and growth of the Δ*lasR* strain can only occur on products released by the wild type. To do this, we used choline as a carbon source (see Fig. 5A for the schematic of the experimental strategy). Both free choline and choline incorporated into eukaryotic lipids are abundant in infections (55, 56). For these experiments, we engineered a Δ*lasR* strain that lacked *betA* and *betB*, which encode a choline oxidase and betaine aldehyde dehydrogenase (57), respectively, which are essential for the catabolism of choline as a growth substrate. To test if the wild-type-secreted products could support Δ*lasR*Δ*betAB* growth in choline medium, we collected supernatant from wild-type cultures grown on 20 mM choline and assessed the growth of Δ*lasR*Δ*betAB* in the supernatant diluted 1:1 in fresh medium (Fig. 5A). Supernatants from the wild-type culture supported a fourfold increase in Δ*lasR*Δ*betAB* density on average over the same strain in fresh medium (Fig. 5B). Furthermore, growth of the Δ*lasR*Δ*betAB* strain was no longer observed if *tctED* (necessary for growth on citrate) (Fig. S3B) was also deleted, as we observed a 1.4-fold growth enhancement for the Δ*lasR*Δ*betAB*Δ*tctED* strain in supernatant relative to fresh medium, which was significantly less than the boost observed for the Δ*lasR*Δ*betAB* strain (Fig. 5B). Supplementing the supernatants with glucose supported greater relative growth than the supernatant alone for both the Δ*lasR*Δ*betAB* and the Δ*lasR*Δ*betAB*Δ*tctED* strains, whereas citrate supplementation supported greater relative growth of the Δ*lasR*Δ*betAB* strain compared to supernatant alone, but not for the Δ*lasR*Δ*betAB*Δ*tctED* strain, as expected (Fig. 5B). Because supplementation of glucose or citrate supported additional growth, the supernatants were not inherently restrictive nor limiting aside from carbon. The *tctED*-dependent growth enhancement observed for the Δ*lasR*Δ*betAB* strain in the presence of wild-type-secreted factors is consistent with a model of citrate cross-feeding from wild-type to Δ*lasR* cells in co-culture.

### Citrate cross-feeding benefits LasR− strains during intraspecies co-cultures

**Fig 6:**
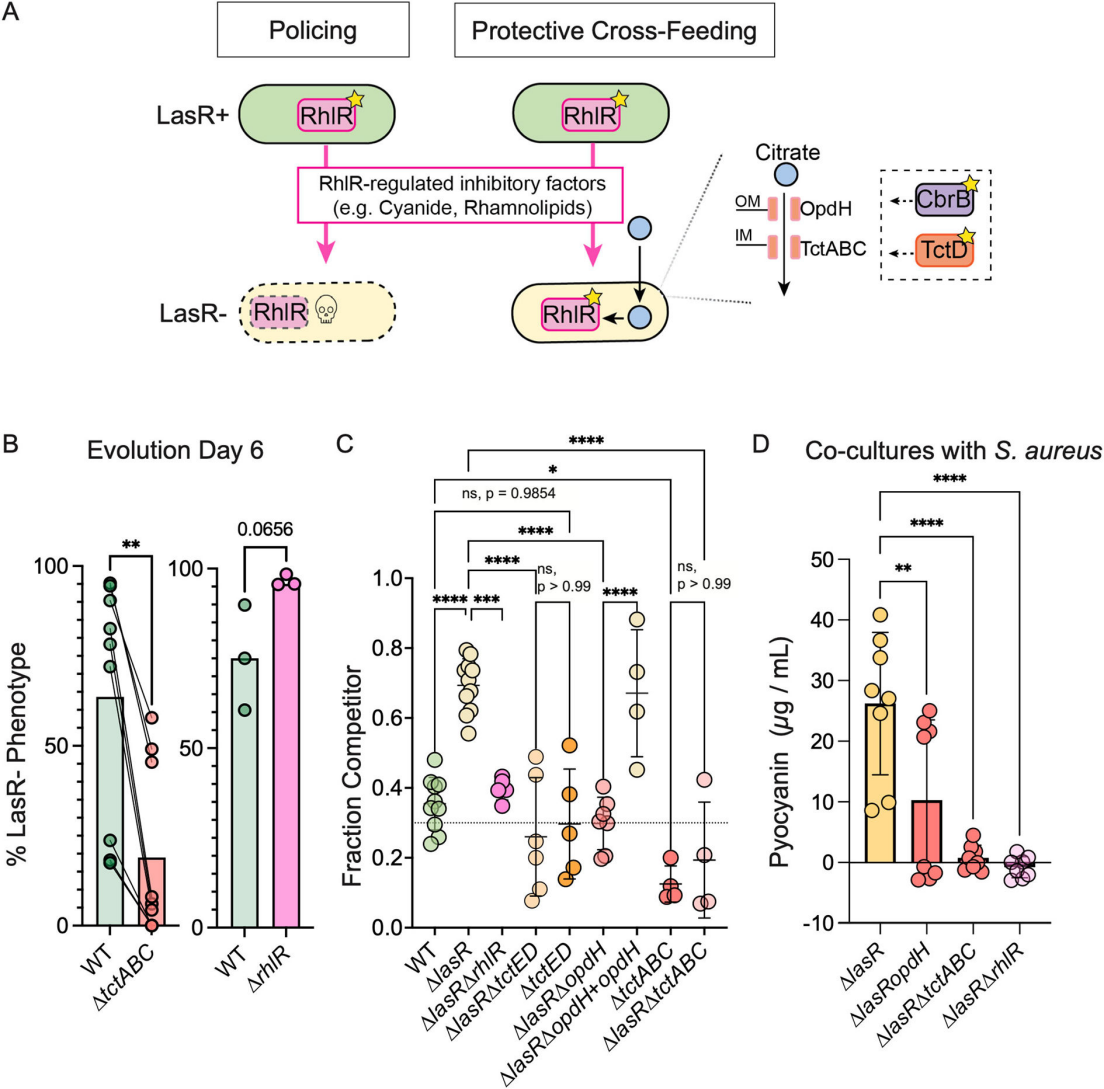
Citrate uptake and RhlR activation impact *lasR* mutant fitness. (A) Model illustrating proposed RhlR-dependent interactions in the absence and presence of cross-feeding. Activity of the quorum-sensing regulator RhlR (pink rectangle with a star indicating activation) reduces sensitivity to RhlR-regulated exoproducts. LasR+ strains (green oval) with active RhlR can “police,” or restrict the rise of LasR− strains (beige oval) due to differences in RhlR activity. However, citrate cross-feeding from wild type (or other neighboring organisms) and uptake by LasR− strains via the OpdH outer membrane (OM) porin and TctABC inner membrane (IM) transporter under TctD (orange rectangle) transcriptional control and CbrB (purple rectangle) post-transcriptional control promote RhlR-dependent fitness and survival of LasR− strains in a process we term “protective cross-feeding.” (B) Percent of population with LasR loss-of-function phenotypes at day 6 after two passages in LB over an experimental evolution originating from a PA14 wild-type, Δ*tctABC*, or Δ*rhlR* ancestral strains. The data presented for the Δ*rhlR* strain are from our previously published report (29) utilizing the same evolution regime. Statistical significance was determined via unpaired *t*-tests. (C) Competitive fitness of Δ*lasR*, Δ*lasR*Δ*rhlR*, Δ*lasRΔtctED*, Δ*tctED*, Δ*lasR*Δ*opdH*, Δ*lasR*Δ*opdH + opdH*, Δ*tctABC*, and Δ*lasR*Δ*tctABC* competitor strains after 16-h growth on LB agar as colony biofilms when initiated at 30% of the starting inoculum (dotted line) with the neutrally tagged wild type (*att::lacZ*) making up the difference. Statistical significance was determined via ordinary one-way ANOVA with Šidák’s multiple comparisons test. (D) Pyocyanin levels for the indicated *lasR* mutants in co-culture with *Staphylococcus aureus*. Statistical significance was determined via ordinary one-way ANOVA with Šidák’s multiple comparisons test. For all tests, *P value < 0.05; **P value < 0.006; ****P value = 0.0001; ****P value < 0.0001; and ns, not significant—*P* value as indicated.

In light of the findings that (i) secreted citrate is taken up by Δ*lasR* but not wild-type cells via CbrAB and TctED-dependent mechanisms (Fig. 2C), (ii) that in Δ*lasR* cells, citrate induces RhlR activity (Fig. 4D and E), and (iii) published studies indicating the lack of RhlR activity impairs the survival of Δ*lasR* cells due to low levels of RhlR-regulated resistance against QS-regulated exoproducts released in a process termed “policing” (Fig. 6A) (58), we hypothesized that citrate uptake may play a protective role against RhlR-mediated strain-strain inhibition by activating RhlR in LasR− strains—a model we term “protective cross-feeding” (Fig. 6A). Using the experimental system which showed that CbrAB was necessary for the rise of LasR− strains *in vitro* (29), we tested the importance of CbrB-dependent citrate uptake for *lasR* mutant selection as a way to assess fitness. Given *tctABC* is likely under CbrB-Crc control and is required for growth on citrate (Fig. S3B), we chose the Δ*tctABC* strain to assess this question. We monitored the rise of LasR− strains in a Δ*tctABC* strain, which showed that LasR− lineages arose more slowly reaching significantly lower proportions of the population by Day 6 when citrate uptake was hindered (Fig. 6B). Consistent with studies describing a RhlR-dependent mechanism to restrict LasR− strain frequency, our previously published data utilizing the same evolution regime depict a more rapid rise of LasR− strains when initiated with a Δ*rhlR* strain (Fig. 6B).

Given RhlR has been implicated in the competitive fitness of *lasR* mutant clinical isolates (59, 60), and the presence of LasR+ cells induces RhlR activity in LasR− strains in a TctED-dependent manner (Fig. 4C), we directly assessed the competitive fitness of the Δ*lasR*Δ*rhlR* strain alongside those deficient in citrate uptake relative to the Δ*lasR* strain. We chose to assess the competitive fitness when LasR− strains were initiated at 30% of the population to be consistent with prior work, which included an assessment of co-culture outcomes across a range of ratios (20). Consistent with the observed rise of LasR− strains over the course of serial passaging and previous demonstrations (20, 39), the Δ*lasR* strain was more fit than the wild type in competition assays with a “wild-type” strain harboring a constitutive *lacZ* marker. The Δ*lasR* strain showed a significant increase in competitive fitness, with the percentage of the Δ*lasR* competitor rising from 30% of the population at the start of the experiment to ∼70% after 16 h, while the untagged wild-type control remained close to the starting fraction (0.3) when grown with the tagged strain (Fig. 6C). The Δ*lasR*Δ*rhlR* strain, however, was significantly less fit than the Δ*lasR* strain, with the fraction of Δ*lasR*Δ*rhlR* cells remaining close to the fraction of the starting inoculum (Fig. 6C). Interestingly, the fitness advantage of the Δ*lasR* mutant was not observed in the Δ*lasR*Δ*tctED*, Δ*lasR*Δ*tctABC*, or Δ*lasR*Δ*opdH* strains with altered citrate consumption (Fig. 6C), and the competitive fitness of the Δ*lasR*Δ*opdH* strain was restored when *opdH* was complemented back onto the chromosome (Δ*lasR*Δ*opdH + opdH*) (Fig. 6C). We did not observe any deficit in growth yield or rate between *lasR* mutant derivatives when grown as monocultures on LB, suggesting the fitness cost may be specific to co-culture (Fig. S3D). In contrast to the TctED-dependent competitive fitness advantage of LasR− strains, Δ*tctED* in the LasR+ background did not have a fitness cost, whereas loss of TctABC did impact LasR+ fitness as well (Fig. 6C). Because RhlR is induced by LasR− cells in co-culture with wild type (Fig. 4B and C; Fig. S5), and RhlR is important for the competitive fitness of the Δ*lasR* strain, those cells that do not induce RhlR in co-culture may be outcompeted.

### Citrate cross-feeding also benefits LasR− strains in co-culture with *Staphylococcus aureus*

Previous studies showed that RhlR-regulated factors also contribute to *P. aeruginosa-S. aureus* interactions (as reviewed in reference 61). An in-depth analysis of the *P. aeruginosa-Staphylococcus aureus* interactions by Zarrella and Khare showed that wild-type *S. aureus* strains had supernatant citrate concentrations in the 100–400 µM range and that *S. aureus* supernatants induced *opdH* in *P. aeruginosa* (34). Furthermore, prior results were suggestive of increased pyocyanin production by *lasR* mutants when adjacent to *S. aureus* (62). Thus, we sought to determine if citrate uptake by Δ*lasR* strains contributed to these interactions as well. *S. aureus* was spread plated on tryptic soy agar (TSA) medium to initiate an even lawn, spot inoculated with *P. aeruginosa* to initiate colony biofilm growth, and then co-incubated. We found that while the Δ*lasR* strain showed strong blue-green pigmentation attributable to phenazines, the Δ*lasR*Δ*rhlR,* Δ*lasR*Δ*tctABC*, and Δ*lasR*Δ*opdH* strains did not. Upon quantification, we found significantly less pyocyanin when *S. aureus* was co-cultured with *lasR* mutant derivatives lacking the ability to consume citrate or activate RhlR (Fig. 6D). Together, these data show that citrate uptake by *P. aeruginosa* contributes to RhlR-dependent interactions between genotypes of *P. aeruginosa* and across species in *P. aeruginosa-S. aureus* interactions.

## DISCUSSION

Our data suggest that citrate cross-feeding occurs between *P. aeruginosa* LasR+ strains and *lasR* mutants, which are frequently co-isolated with LasR+ strains (6, 11), and that this interaction induces RhlR signaling and phenazine production in the latter cell type. Citrate induction of RhlR-regulated virulence factors by LasR− strains might offer one explanation for the association of LasR− strains with worse disease outcomes in CF. Given the polymicrobial nature of CF infections (37), there are likely multiple sources of citrate in CF airways. To this point, we also demonstrated citrate cross-feeding between LasR− *P. aeruginosa* and *S. aureus*. In *P. aeruginosa* and *S. aureus* co-infections, which are common in CF, citrate produced by *S. aureus* may markedly enhance the virulence factor production by the prevalent LasR− strains thereby causing more severe lung disease (36, 38, 63).

We found that both *P. aeruginosa* wild type and *lasR* mutants are capable of citrate secretion (Fig. 2D). The secretion of citrate may result from “noisy metabolism” that promotes metabolite leakage (64), or shifts in iron availability that induce its release as a metal scavenging molecule (65) or a means to combat iron overload as with the IceT transport protein in *Salmonella* (66). We have previously reported that the siderophore pyochelin promotes citrate release by wild-type cells (20), suggesting secretion may relate to iron availability or the signaling capacity of pyochelin itself (67). While pyochelin supplementation is not necessary for citrate secretion, production of this siderophore by LasR− strains is required for co-culture interactions, at least on some media (20).

LasR− strains have increased CbrA-CbrB activity relative to LasR+ strains (29), but CbrB did not have striking effects on citrate secretion (Fig. 2D). Citrate utilization depended on the CbrAB two-component system (Fig. 2B), and elevated CbrA-CbrB does appear to explain increased consumption of citrate by LasR− cells compared to LasR+ counterparts (Fig. 2C). We previously showed that co-culture of LasR+ and LasR− strains stimulates RhlR activity and RhlR-controlled pyocyanin production (20), and here we demonstrated that this stimulation required both CbrB and citrate uptake via the outer membrane porin OpdH and the membrane transporter TctABC (Fig. 4C and F). Citrate induces these transporters via the TctED two-component system (33, 44, 45) (Fig. 3A; Fig. S3A and C). Wild-type *P. aeruginosa* cultures can accumulate upwards of 300 µM citrate, and we observed significant increases in TctD-dependent *opdH* promoter activity in co-culture (Fig. 3C). As shown by Underhill et al. (33), OpdH was not required for growth on millimolar concentrations of citrate as a sole carbon source (Fig. S3B), suggesting that other porins can take up citrate when it is present at high levels (68). Our data may suggest a greater role for OpdH in citrate transport when it is at lower (e.g., micromolar) concentrations. From the periplasm, tricarboxylate transporters can bring citrate across the inner membrane (33). Our data suggest that once citrate is taken up by LasR− cells, it induces RhlR-dependent transcriptional activity.

When activated by RhlI-synthesized C4HSL, RhlR is important for competitive fitness in strain PAO1 among other strains (58, 59, 69). Here, we show a similar requirement for RhlR in the PA14 Δ*lasR* strain when co-cultured with the wild type (Fig. 6C). Citrate induction of RhlI/RhlR-dependent signaling may increase *lasR* mutant fitness in LasR+/LasR− co-cultures by increasing its resistance to RhlR-controlled exoproducts (e.g., hydrogen cyanide, rhamnolipids, or other secreted factors (58, 70) (Fig. 6A). The induction of *rhlI* promoter activity required tricarboxylate transport machinery (Fig. 4C and E), and the differential citrate uptake likely explains why citrate induces the activity of RhlR in several LasR− but not LasR+ clinical strains (20). Consistent with this model, we observed a fitness deficit in *lasR* mutants lacking tricarboxylate regulatory components, uptake machinery, or RhlR itself when grown in the presence of wild type (Fig. 6C), but not in isolation (Fig. S3D).

The mechanism by which citrate stimulates RhlR is not yet known. Our data suggest that citrate does not directly activate RhlR as we showed that RhlI is required for citrate-induced *rhlI* promoter activity (Fig. 4D). This is consistent with prior studies that show growth in LB with citrate leads to higher levels of RhlI-HA, even when expressed under a regulated promoter (20). Thus, we speculate that changes in RhlI proteolysis support activation of RhlR perhaps via ClpXP or Lon proteases given that RhlI is a target of ClpXP (71, 72) and RhlI-HA levels are elevated but non-inducible in a *clpX* disruption mutant (20). The effects of ClpX on RhlI may also be indirect. For example, pyochelin biosynthetic proteins are also a predicted target of ClpXP proteolysis (73), and pyochelin can stimulate citrate release as discussed above (20). Additional studies are needed to understand the effects of citrate on ClpXP. Although biochemical assays with purified ClpP protein indicate that citrate induces activity for one of two ClpP isomers (74), studies in live cells suggest co-culture with *S. aureus*, where citrate cross-feeding has been demonstrated, leads to decreases in *P. aeruginosa* ClpXP activity (73). Decreased ClpXP activity is predicted to lead to an increase in RhlI protein and RhlR activity, which may explain the increased pyocyanin production presented here.

We observed TctED-dependent interactions for *S. aureus* and the wild-type *P. aeruginosa* with *lasR* loss-of-function mutants (Fig. 6D), suggesting that citrate cross-feeding is necessary, but we do not address whether it is sufficient to recapitulate the observed microbial interactions. The comparison of intracellular metabolomes between LasR− and LasR+ cells across strain backgrounds and medium type suggests differences in other metabolites as well (Fig. 1A). Thus, citrate may not be the sole metabolite exchanged between LasR+ and LasR− strains. Furthermore, the uptake of additional metabolites is predicted to be under CbrB-Crc control, and these may influence RhlR or other virulence-associated pathways. Many metabolites, such as pyruvate, lactate, and acetate, which are commonly secreted as a result of cell death, overflow metabolism, or the Warburg effect (43, 75, 76), require CbrB for consumption and/or metabolism (77). Metabolite exchange can allow new microbial inhabitants to thrive, alter ecosystem structure, and give rise to emergent properties or functions specific to cells when at high densities. Additional work will be required to elucidate how the interactions described here may shape community structure in evolving *P. aeruginosa* populations and its interactions with neighboring cells.

The higher intracellular citrate observed in LasR− strains in this study parallels metabolomics analysis that identified elevated citrate concentrations in a strain that cannot produce autoinducers important for quorum-sensing activity (i.e., Δ*lasI*Δ*rhlI*) (28). Collectively, this suggests that differences in citrate are linked to quorum-sensing activity broadly. The distinct metabolic states induced by quorum-sensing activity highlight the potential for similar cross-feeding interactions to occur within genetically identical or clonal populations where distinct clusters of cells with differential quorum-sensing activity exist. Heterogeneity in quorum-sensing signaling has been well documented (78–80), lending support to this possibility, and extending the implications beyond genetically distinct cells to identical ones with distinct expression profiles. Beyond *P. aeruginosa*, many other cell types (host and microbial) secrete citrate. Thus, the cross-feeding interactions described here likely extend to those between other microbes and host cells where virulence factor production is differentially stimulated between LasR− and LasR+ strains. Thus, these studies add to the growing list of examples of how metabolic heterogeneity within a population can promote resilience and hinder the eradication of infections in part through inter- and intra-species cross-feeding (81–84).

## MATERIALS AND METHODS

### Strains and growth conditions

The strains used in this study are listed in Table S2. *P. aeruginosa* strains were maintained on LB (10 g tryptone, 5 g yeast extract, and 5 g NaCl per L) with 1.5% agar. *S. aureus* was maintained in TSB with 1.5% agar. Yeast strains for cloning were maintained on YPD (10 g yeast extract, 20 peptone, and 20 g dextrose per L) with 2% agar at 30°C. Planktonic bacterial cultures (5 mL) were grown on a roller drum at 37°C in 13-mm borosilicate tubes.

### Strain construction

Plasmids were constructed using a *Saccharomyces cerevisiae* recombination technique described previously (85). The construct for constitutive mKate2 fluorescence was constructed as described in Kasetty et al. with two tandem copies of codon-optimized *mKate2* under a synthetic tac promoter (86). Plasmid candidates were selected by restriction digest, and successful candidate plasmids were sequenced for verification of the insert. In frame-deletions, complementation constructs, and integrated promoter fusions were introduced into *P. aeruginosa* by conjugation via S17/lambda pir *E. coli*. Merodiploids were selected by drug resistance, and double recombinants were obtained by sucrose counter-selection and genotype screening by PCR.

### Metabolomics

Strains were grown in 5 mL of LB for 16 h from single colonies taken from freshly struck strains on LB plates, and 5 µL of the overnight culture was used to initiate colony biofilm growth on LB agar or artificial sputum medium agar made as described in reference 39. For each replicate, four plates with 18 colony biofilms each were pooled during collection after 16 h of growth at 37°C. Colonies were scraped from the agar surface using a rubber policeman, deposited in a 1.5-mL tube, briefly pelleted, stored at −80°C, and sent to Metabolon (Morrisville, NC, USA) for metabolomics analysis. Five replicates were collected for each sample/condition. Raw counts were scaled and imputed, and then normalized using the total raw counts across all metabolites by sample. Pareto-normalized metabolite counts were used as input for principal component analysis using the R package ggfortify (87). The ggplot2 and ggprism R packages were used to visualize the differential metabolite data using R (version 4.2.1) (88, 89). Only metabolites detected across all samples in a comparison were included in volcano plots.

### Citrate quantification

For extracellular citrate measurements, 5 mL of LB was inoculated with a single colony, grown for 16 h, pelleted by centrifugation for 10 min at 5,000 RPM in 15 mL conical tubes, and filtered through a 0.22 µm pore-size polycarbonate filter. Citrate was measured as previously (20) using 1/2 reactions in a commercially available Megazyme citric acid enzymatic kit (cat. K-CITR). A citrate standard was included in every assay. The citrate concentration is reported relative to the cellular density (OD_600_) measured after 16 h of growth at 37°C on a roller drum where indicated. For citrate consumption assays, a 16-h LB culture was adjusted to an OD_600_ of 1.5 in 2 mL total volume of LB and incubated at 37°C for 10 min after which 2 mM citrate was added. Cultures were incubated statically at 37°C for 24 h with Parafilm covering the uncapped tube opening to allow gas exchange and reduce evaporation. After incubation, cells were pelleted for 10 min at 13,000 RPM and filtered as previously described for assessment in the enzymatic assay as described above. Each experiment was repeated on at least three separate days.

### Growth assays

Growth was assessed in M63 base [10 g (NH_4_)_2_SO_4_, 15 g KH_2_PO_4_, and 35 g K_2_HPO_4_ per liter with 1 mM MgSO_4_) containing 10 mM citrate in two distinct ways. Data shown in Fig. 2B and Fig. S4B were collected using the method described in reference 29. In brief, 5 mL LB cultures grown for 16 h were adjusted to an OD_600_ of 1 in LB, and a 250-µL aliquot of the normalized culture was added to 5 mL of fresh M63 base containing 10 mM disodium citrate (Acros) as a sole carbon source. The OD_600_ was monitored using a Spectronic 20D+ (Spec20) during growth on a roller drum at 37°C. Growth on LB was monitored in the same way. Growth reported in Fig. S3B was performed by inoculating single colonies from freshly struck LB plates into 5 mL of M63 medium with 10 mM disodium citrate (Acros). The density (OD_600_) was measured in a 1 cm cuvette with a 1:10 dilution after 24 h of incubation on a roller drum at 37°C. Each experiment was repeated on at least three separate days.

### Beta-galactosidase quantification

Cells with a promoter fusion to *lacZ*–GFP integrated at the *att* locus were grown in 5 mL cultures of LB at 37°C for 16 h on a roller drum. The cultures were diluted to a starting OD_600_ of 1 in LB for use in the experimental set up. For assessments as colony biofilms (as in Fig. 3A and B; Fig. 4D and E), a 5-µL aliquot of each normalized culture was spotted onto LB agar plates with the indicated concentration of disodium citrate (pH 7). After 24 h at 37°C, the colony biofilms were cored with the back of a P1000 pipette tip, the cells were re-suspended in 500 µL of LB by vigorous shaking on a Genie Disrupter for 5 min as previously described (20), and a cell suspension normalized to an OD_600_ of 0.5 was used as input into the β-gal assay. β-gal activity was measured as described by Miller using the following equation: Miller unit = 1,000 × [OD_420_ − (1.75 × OD_550_)]/(time × volume × OD_600_) , where time is reported in minutes and volume in milliliters (90). For co-culture induction assays, a 250-µL aliquot of normalized (OD_600_ = 1) culture was added to 5 mL of fresh LB to initiate monocultures and incubated on a roller drum for 6 h at 37°C. For co-cultures, the inoculation was initiated in an 8:2 ratio of the PA14 *att*:P*tac*-mKate strain to the Δ*lasR* derivative containing the designated promoter fusion. To do this, we added a 200-µL aliquot of the OD-adjusted (OD_600_ = 1) PA14 *att:*P*tac*-mKate strain and a 50-µL aliquot of the OD-adjusted *lasR* mutant to 5 mL of LB for comparison with the monocultures, where 250 µL of OD_600_ = 1 normalized cells was added. After 6 h, we collected the cultures and adjusted the optical density to 0.5 for use in the β-gal assay. An appropriate dilution was plated for each co-culture onto LB plates containing 150 µg/mL 5-bromo-4-chloro-3-indolyl-D-galactopyranoside (X-Gal) using glass beads, and the plates were incubated at 37°C until blue colonies were visible (∼24 h). To calculate the relative β-Gal activity, the OD_600_ value in the Miller unit equation was multiplied by the fraction containing the *lacZ* marker as indicated by blue:white colony counts. Each experiment was repeated in at least four independent experiments.

### Pyocyanin quantification

Overnight LB cultures were normalized in LB to an OD_600_ = 1. For monocultures, a 3-µL aliquot of OD-adjusted cultures was spotted in four replicate wells of a 96-well plate in which each well was pre-filled with 200 µL of LB agar. For co-cultures, 700 µL of the Δ*phz* strain was mixed with 300 µL of the indicated *lasR* mutant, vortexed, and then a 3-µL aliquot of the mixed culture was plated in four replicate wells of the same 96-well plate as the monocultures on top of the LB agar. After 16 h of incubation at 37°C, the 96-well agar plates were placed at −80°C until extraction. For pyocyanin extraction, two agar plugs from two replicate wells of the 96-well plate were placed in a 2 mL Eppendorf tube with 500 µL of chloroform and agitated on a Genie Disruptor (Zymo) for 2 min. After which 200 µL of the bottom chloroform layer was placed into a fresh 1.5 mL tube, and the chloroform extraction was repeated with 500 µL of fresh chloroform. Another 300 µL of the bottom layer was added to the previous 200 µL aliquot. To the chloroform layer, 500 µL of 0.2 N HCl was added to acidify the sample turning the pyocyanin from blue to pink, and the optical density was measured at 520 nm alongside an acidified pyocyanin standard to determine the concentration in µg/mL for each strain by interpolation. The pyocyanin extracted was reported per colony biofilm (or agar plug).

### Flow cytometry

Strains with the promoter from the RhlR-regulated gene *rhlI* fused to green fluorescent protein and the *lacZ* gene were grown in monoculture or in co-culture with a “wild-type” strain constitutively expressing the mKate2 fluorophore under a synthetic *tac* promoter, referred to as the PA14 att:P*tac*-mKate strain. Overnight LB cultures of the PA14 att:P*tac*-mKate strain and the designated *lasR* mutants with the *lacZ*–GFP fusion integrated at the Tn7 *att* site were adjusted to an OD_600_ of 1. For monocultures, a 250-µL aliquot was added to 5 mL fresh LB. For co-cultures, 200 µL of the PA14 att:P*tac*-mKate and 50 µL of the designated *lasR* mutant were added to 5 mL of fresh LB. After 6 h of incubation on a roller drum at 37°C, a 500-µL aliquot of the subculture was pelleted for 5 min at 13,000 RPM, resuspended in 500 µL of PBS + 0.01% Triton X-100, diluted 100-fold in PBS + 0.01% Triton X-100, and diluted again 1:1 in PBS without detergent. The diluted cells were placed on ice until processed by flow cytometry. The data were collected by Beckman Coulter Cytoflex S and analyzed with FlowJo version 10.8.1. In short, single-cell gating was done using FSC versus SSC, and cells without mKate expression were gated using the ECD channel. GFP expression was quantified by fluorescein isothiocyanate (FITC)median fluorescence intensity of the single-cell, mKate-negative populations.

### Syntrophic growth assays on choline

Single colonies of PA14 wild type were inoculated into a 250 mL baffled flask containing 100 mL of M63 base with 20 mM choline chloride. After 48 h on a shaker (240 RPM) at 37°C, cultures were pelleted at max speed for 20 min in 50 mL falcon tubes. The supernatant was filtered through a 0.22-µm pore size polycarbonate filter and stored at 4°C. The supernatant was diluted 1:1 in fresh choline medium with 2 mL per well in a 12-well plate. Colonies of the Δ*lasR*Δ*betAB* or Δ*lasR*Δ*betAB*Δ*tctED* strain from a freshly struck LB plate were re-suspended in water and adjusted to an OD_600_ of 2. The cell re-suspension was used to inoculate the diluted supernatant or fresh choline medium at a starting OD_600_ of 0.05, and growth was monitored after 48 h by CFU count in *n* > 3 experiments and/or OD_600_ in two experiments. Supernatants from experiments collected on at least three distinct days were tested for growth enhancement in at least three separate experiments.

### Competition assays

Competition assays were performed as in reference 20. In summary, strains were grown for 16 h from a single colony in a 5 mL LB culture on a roller drum at 37°C. Based on a 1:10 dilution of the 16 h culture in a 1 cm cuvette, cultures were adjusted to OD_600_ = 1 in the required volume of LB. Density-adjusted competitor strains were mixed with PA14 *att::lacZ* strain in a 7:3 ratio by adding 700 µL of the PA14*att::lacZ* strain with 300 µL of the competitor strain. This ratio was selected to be consistent with prior work, which included the investigation of many ratios for co-culture interactions (20). Following a brief vortex, a 5-µL aliquot of the combined suspension was spotted on LB agar. After 16 h, colony biofilms (and agar) were cored using the back of a sterile P1000 pipette tip, placed in 1.5 mL tubes with 500 µL LB, and shaken vigorously for 5 min using a Genie Disruptor (Zymo). This suspension was diluted in LB, spread on LB plates supplemented with 150 µg/mL X-Gal using glass beads, and incubated at 37°C until blue colonies were visible (∼24 h). The number of blue and white colonies per plate was counted and the final proportions were recorded. Each competition was replicated on four to seven separate days.

### *Staphylococcus aureus* interaction assay

The protocol was based off of reference 91 with modifications. *S. aureus* strain SH1000 was grown in 5 mL of TSB medium at 37°C for 16 h and adjusted to OD_600_ = 0.1 in the required volume of TSB. A 100- or 150-µL aliquot of density-adjusted *S. aureus* culture was spread on a TSA plate with sterile glass beads and allowed to dry in a biosafety cabinet for ∼10 min. *P. aeruginosa* cultures (16 h, 5 mL LB) were adjusted to OD_600_ = 1 in LB medium, and a 5-µL aliquot was spotted onto the lawn of *S. aureus* after the drying period to prevent colony spreading. After 16 h incubation at 37°C, plates were placed at room temperature and imaged daily with a Canon EOS Rebel T6i digital camera. The phenazine pyocyanin was extracted from cored *P. aeruginosa* colonies and the underlying *S. aureus* lawn on Day 3 and quantified as described above. Each experiment was repeated on at least three independent days.

### Experimental evolution

Experimental evolution was performed exactly as described in reference 29. In brief, an LB culture was inoculated with a single colony of the indicated strain, grown for 24 h, and normalized to an OD_600_ of 1 in LB. This normalized culture was used to begin three independent lines with a starting OD_600_ of 0.05. These three lines were passaged every 48 h with 25 µL being transferred to 5 mL of fresh LB medium at each passage. At each time point, a 35–50 µL aliquot at a 10^−5^ dilution was plated onto LB agar, and the percentage of colonies with a sheen LasR− phenotype due to HHQ accumulation was enumerated. Each evolution was repeated three times (with three lines each) and counted by an independent enumerator.

## Data Availability

All data from metabolomics analysis, including raw and normalized counts, are included in Table S1. All materials and data are included in the paper or supplemental material or will be made available in a timely fashion, at reasonable cost, and in limited quantities to members of the scientific community for noncommercial purposes, subject to requirements or limitations imposed by local and/or U.S. Government laws and regulations.

## SUPPLEMENTAL MATERIAL

Supplemental figures mbio.01278-23-s0001.pdf Figures S1-S5.

Table S1 mbio.01278-23-s0002.xlsx Metabolomics data set.

Table S2 mbio.01278-23-s0003.pdf Strains and plasmids.

ASM does not own the copyrights to Supplemental Material that may be linked to, or accessed through, an article. The authors have granted ASM a non-exclusive, world-wide license to publish the Supplemental Material files. Please contact the corresponding author directly for reuse.

## References

[1] Maharjan RP, Seeto S, Ferenci T. 2007. Divergence and redundancy of transport and metabolic rate-yield strategies in a single Escherichia coli population. J Bacteriol 189:2350–2358. doi:10.1128/JB.01414-06 17158684 PMC1899394

[2] Wright NR, Jessop-Fabre MM, Sánchez BJ, Wulff T, Workman CT, Rønnest NP, Sonnenschein N. 2022. Emergence of phenotypically distinct subpopulations is a factor in adaptation of recombinant Saccharomyces cerevisiae under glucose-limited conditions. Appl Environ Microbiol 88:e0230721. doi:10.1128/aem.02307-21 35297727 PMC9004382

[3] Heppner GH. 1984. Tumor heterogeneity. Cancer Res 44:2259–2265. 6372991

[4] Smith EE, Buckley DG, Wu Z, Saenphimmachak C, Hoffman LR, D’Argenio DA, Miller SI, Ramsey BW, Speert DP, Moskowitz SM, Burns JL, Kaul R, Olson MV. 2006. Genetic adaptation by Pseudomonas aeruginosa to the airways of cystic fibrosis patients. Proc Natl Acad Sci U S A 103:8487–8492. doi:10.1073/pnas.0602138103 16687478 PMC1482519

[5] Feltner JB, Wolter DJ, Pope CE, Groleau MC, Smalley NE, Greenberg EP, Mayer-Hamblett N, Burns J, Déziel E, Hoffman LR, Dandekar AA, Winans SC, Diggle S, Goldberg J. 2016. Lasr variant cystic fibrosis isolates reveal an adaptable quorum-sensing hierarchy in Pseudomonas aeruginosa. mBio 7:e01513-16. doi:10.1128/mBio.01513-16 27703072 PMC5050340

[6] Hoffman LR, Kulasekara HD, Emerson J, Houston LS, Burns JL, Ramsey BW, Miller SI. 2009. Pseudomonas aeruginosa lasR mutants are associated with cystic fibrosis lung disease progression. J Cyst Fibros 8:66–70. doi:10.1016/j.jcf.2008.09.006 18974024 PMC2631641

[7] Cramer N, Klockgether J, Wrasman K, Schmidt M, Davenport CF, Tümmler B. 2011. Microevolution of the major common Pseudomonas aeruginosa clones C and PA14 in cystic fibrosis lungs. Environ Microbiol 13:1690–1704. doi:10.1111/j.1462-2920.2011.02483.x 21492363

[8] Cramer N, Klockgether J, Tümmler B. 2023. Microevolution of Pseudomonas aeruginosa in the airways of people with cystic fibrosis. Curr Opin Immunol 83:102328. doi:10.1016/j.coi.2023.102328 37116385

[9] Yang L, Jelsbak L, Marvig RL, Damkiær S, Workman CT, Rau MH, Hansen SK, Folkesson A, Johansen HK, Ciofu O, Høiby N, Sommer MOA, Molin S. 2011. Evolutionary dynamics of bacteria in a human host environment. Proc Natl Acad Sci U S A 108:7481–7486. doi:10.1073/pnas.1018249108 21518885 PMC3088582

[10] Marvig RL, Sommer LM, Molin S, Johansen HK. 2015. Convergent evolution and adaptation of Pseudomonas aeruginosa within patients with cystic fibrosis. Nat Genet 47:57–64. doi:10.1038/ng.3148 25401299

[11] Winstanley C, O’Brien S, Brockhurst MA. 2016. Pseudomonas aeruginosa evolutionary adaptation and diversification in cystic fibrosis chronic lung infections. Trends Microbiol 24:327–337. doi:10.1016/j.tim.2016.01.008 26946977 PMC4854172

[12] Kostylev M, Kim DY, Smalley NE, Salukhe I, Greenberg EP, Dandekar AA. 2019. Evolution of the Pseudomonas aeruginosa quorum-sensing hierarchy. Proc Natl Acad Sci U S A 116:7027–7032. doi:10.1073/pnas.1819796116 30850547 PMC6452656

[13] O’Connor K, Zhao CY, Mei M, Diggle SP. 2022. Frequency of quorum-sensing mutations in Pseudomonas aeruginosa strains isolated from different environments. Microbiology (Reading) 168:12. doi:10.1099/mic.0.001265 PMC10233726 36748632

[14] Wilder CN, Allada G, Schuster M. 2009. Instantaneous within-patient diversity of Pseudomonas aeruginosa quorum sensing populations from cystic fibrosis lung infections. Infect Immun 77:5631–5639. doi:10.1128/IAI.00755-09 19805523 PMC2786440

[15] Whiteley M, Diggle SP, Greenberg EP. 2017. Progress in and promise of bacterial quorum sensing research. Nature 551:313–320. doi:10.1038/nature24624 29144467 PMC5870893

[16] Lesprit P, Faurisson F, Join-Lambert O, Roudot-Thoraval F, Foglino M, Vissuzaine C, Carbon C. 2003. Role of the quorum-sensing system in experimental pneumonia due to Pseudomonas aeruginosa in rats. Am J Respir Crit Care Med 167:1478–1482. doi:10.1164/rccm.200207-736BC 12569080

[17] Pearson JP, Feldman M, Iglewski BH, Prince A. 2000. Pseudomonas aeruginosa cell-to-cell signaling is required for virulence in a model of acute pulmonary infection. Infect Immun 68:4331–4334. doi:10.1128/IAI.68.7.4331-4334.2000 10858254 PMC101761

[18] Rumbaugh KP, Griswold JA, Iglewski BH, Hamood AN. 1999. Contribution of quorum sensing to the virulence of Pseudomonas aeruginosa in burn wound infections. Infect Immun 67:5854–5862. doi:10.1128/IAI.67.11.5854-5862.1999 10531240 PMC96966

[19] Wu H, Song Z, Givskov M, Doring G, Worlitzsch D, Mathee K, Rygaard J, Høiby N. 2001. Pseudomonas aeruginosa mutations in lasi and rhli quorum sensing systems result in milder chronic lung infection. Microbiology 147:1105–1113. doi:10.1099/00221287-147-5-1105 11320114

[20] Mould DL, Botelho NJ, Hogan DA. 2020. Intraspecies signaling between common variants of Pseudomonas aeruginosa increases production of quorum-sensing-controlled virulence factors. mBio 11:e01865-20. doi:10.1128/mBio.01865-20 32843558 PMC7448281

[21] Cugini C, Morales DK, Hogan DA. 2010. Candida albicans-produced farnesol stimulates Pseudomonas quinolone signal production in LasR-defective Pseudomonas aeruginosa strains. Microbiology (Reading) 156:3096–3107. doi:10.1099/mic.0.037911-0 20656785 PMC3068698

[22] Hennemann LC, LaFayette SL, Malet JK, Bortolotti P, Yang T, McKay GA, Houle D, Radzioch D, Rousseau S, Nguyen D. 2021. LasR-deficient Pseudomonas aeruginosa variants increase airway epithelial mICAM-1 expression and enhance neutrophilic lung inflammation. PLoS Pathog. 17:e1009375. doi:10.1371/journal.ppat.1009375 33690714 PMC7984618

[23] LaFayette SL, Houle D, Beaudoin T, Wojewodka G, Radzioch D, Hoffman LR, Burns JL, Dandekar AA, Smalley NE, Chandler JR, Zlosnik JE, Speert DP, Bernier J, Matouk E, Brochiero E, Rousseau S, Nguyen D. 2015. Cystic fibrosis-adapted Pseudomonas aeruginosa quorum sensing lasR mutants cause hyperinflammatory responses. Sci Adv 1:6. doi:10.1126/sciadv.1500199 PMC4597794 26457326

[24] Goo E, An JH, Kang Y, Hwang I. 2015. Control of bacterial metabolism by quorum sensing. Trends Microbiol. 23:567–576. doi:10.1016/j.tim.2015.05.007 26072043

[25] An JH, Goo E, Kim H, Seo YS, Hwang I. 2014. Bacterial quorum sensing and metabolic slowing in a cooperative population. Proc Natl Acad Sci U S A 111:14912–14917. doi:10.1073/pnas.1412431111 25267613 PMC4205598

[26] Goo E, Majerczyk CD, An JH, Chandler JR, Seo Y-S, Ham H, Lim JY, Kim H, Lee B, Jang MS, Greenberg EP, Hwang I. 2012. Bacterial quorum sensing, cooperativity, and anticipation of stationary-phase stress. Proc Natl Acad Sci U S A 109:19775–19780. doi:10.1073/pnas.1218092109 23150539 PMC3511722

[27] D’Argenio DA, Wu M, Hoffman LR, Kulasekara HD, Déziel E, Smith EE, Nguyen H, Ernst RK, Larson Freeman TJ, Spencer DH, Brittnacher M, Hayden HS, Selgrade S, Klausen M, Goodlett DR, Burns JL, Ramsey BW, Miller SI. 2007. Growth phenotypes of Pseudomonas aeruginosa lasR mutants adapted to the airways of cystic fibrosis patients. Mol Microbiol 64:512–533. doi:10.1111/j.1365-2958.2007.05678.x 17493132 PMC2742308

[28] Davenport PW, Griffin JL, Welch M. 2015. Quorum sensing is accompanied by global metabolic changes in the opportunistic human pathogen Pseudomonas aeruginosa. J Bacteriol 197:2072–2082. doi:10.1128/JB.02557-14 25868647 PMC4438216

[29] Mould DL, Stevanovic M, Ashare A, Schultz D, Hogan DA. 2022. Metabolic basis for the evolution of a common pathogenic Pseudomonas aeruginosa variant. Elife 11:e76555. doi:10.7554/eLife.76555 35502894 PMC9224983

[30] Sonnleitner E, Sorger-Domenigg T, Madej MJ, Findeiss S, Hackermüller J, Hüttenhofer A, Stadler PF, Bläsi U, Moll I. 2008. Detection of small RNAs in Pseudomonas aeruginosa by RNomics and structure-based bioinformatic tools. Microbiology (Reading) 154:3175–3187. doi:10.1099/mic.0.2008/019703-0 18832323

[31] Malecka EM, Bassani F, Dendooven T, Sonnleitner E, Rozner M, Albanese TG, Resch A, Luisi B, Woodson S, Bläsi U. 2021. Stabilization of Hfq-mediated translational repression by the co-repressor CRC in Pseudomonas aeruginosa. Nucleic Acids Res. 49:7075–7087. doi:10.1093/nar/gkab510 34139006 PMC8266614

[32] Sonnleitner E, Prindl K, Bläsi U. 2017. The Pseudomonas aeruginosa CrcZ RNA interferes with Hfq-mediated riboregulation. PLOS One 12:e0180887. doi:10.1371/journal.pone.0180887 28686727 PMC5501646

[33] Underhill SAM, Cabeen MT. 2022. Redundancy in citrate and Cis-aconitate transport in Pseudomonas aeruginosa. J Bacteriol 204:e0028422. doi:10.1128/jb.00284-22 36321838 PMC9765132

[34] Zarrella TM, Khare A. 2022. Systematic identification of molecular mediators of interspecies sensing in a community of two frequently coinfecting bacterial pathogens. PLoS Biol. 20:e3001679. doi:10.1371/journal.pbio.3001679 35727825 PMC9249247

[35] Fischer AJ, Singh SB, LaMarche MM, Maakestad LJ, Kienenberger ZE, Peña TA, Stoltz DA, Limoli DH. 2021. Sustained coinfections with Staphylococcus aureus and Pseudomonas aeruginosa in cystic fibrosis. Am J Respir Crit Care Med 203:328–338. doi:10.1164/rccm.202004-1322OC 32750253 PMC7874317

[36] Sagel SD, Gibson RL, Emerson J, McNamara S, Burns JL, Wagener JS, Ramsey BW, Inhaled Tobramycin in Young Children Study Group, Cystic Fibrosis Foundation Therapeutics Development Network. 2009. Impact of Pseudomonas and Staphylococcus infection on inflammation and clinical status in young children with cystic fibrosis. J Pediatr 154:183–188. doi:10.1016/j.jpeds.2008.08.001 18822427 PMC2654617

[37] Cystic Fibrosis Foundation. 2021. Cystic fibrosis foundation patient registry 2021 annual data report

[38] Limoli DH, Yang J, Khansaheb MK, Helfman B, Peng L, Stecenko AA, Goldberg JB. 2016. Staphylococcus aureus and Pseudomonas aeruginosa co-infection is associated with cystic fibrosis-related diabetes and poor clinical outcomes. Eur J Clin Microbiol Infect Dis 35:947–953. doi:10.1007/s10096-016-2621-0 26993289

[39] Clay ME, Hammond JH, Zhong F, Chen X, Kowalski CH, Lee AJ, Porter MS, Hampton TH, Greene CS, Pletneva EV, Hogan DA. 2020. Pseudomonas aeruginosa lasR mutant fitness in microoxia is supported by an Anr-regulated oxygen-binding hemerythrin. Proc Natl Acad Sci U S A 117:3167–3173. doi:10.1073/pnas.1917576117 31980538 PMC7022198

[40] Palmer KL, Mashburn LM, Singh PK, Whiteley M. 2005. Cystic fibrosis sputum supports growth and cues key aspects of Pseudomonas aeruginosa physiology. J Bacteriol 187:5267–5277. doi:10.1128/JB.187.15.5267-5277.2005 16030221 PMC1196007

[41] Schuster M, Lostroh CP, Ogi T, Greenberg EP. 2003. Identification, timing, and signal specificity of Pseudomonas aeruginosa quorum-controlled genes: a transcriptome analysis. J Bacteriol 185:2066–2079. doi:10.1128/JB.185.7.2066-2079.2003 12644476 PMC151497

[42] Nouwens AS, Beatson SA, Whitchurch CB, Walsh BJ, Schweizer HP, Mattick JS, Cordwell SJ. 2003. Proteome analysis of extracellular proteins regulated by the las and rhl quorum sensing systems in Pseudomonas aeruginosa PAO1. Microbiology (Reading) 149:1311–1322. doi:10.1099/mic.0.25967-0 12724392

[43] Sasnow SS, Wei H, Aristilde L. 2016. Bypasses in intracellular glucose metabolism in iron-limited Pseudomonas putida. Microbiologyopen 5:3–20. doi:10.1002/mbo3.287 26377487 PMC4767421

[44] Tamber S, Maier E, Benz R, Hancock REW. 2007. Characterization of OpdH, a Pseudomonas aeruginosa porin involved in the uptake of tricarboxylates. J Bacteriol 189:929–939. doi:10.1128/JB.01296-06 17114261 PMC1797325

[45] Taylor PK, Zhang L, Mah TF. 2019. Loss of the two-component system TctD-TctE in Pseudomonas aeruginosa affects biofilm formation and aminoglycoside susceptibility in response to citric acid. mSphere 4:e00102-19. doi:10.1128/mSphere.00102-19 30842268 PMC6403454

[46] Sonnleitner E, Valentini M, Wenner N, Haichar F el Z, Haas D, Lapouge K. 2012. Novel targets of the CbrAB/CRC carbon catabolite control system revealed by transcript abundance in Pseudomonas aeruginosa. PLOS One 7:e44637. doi:10.1371/journal.pone.0044637 23115619 PMC3480352

[47] Corona F, Reales-Calderón JA, Gil C, Martínez JL. 2018. The development of a new parameter for tracking post-transcriptional regulation allows the detailed map of the Pseudomonas aeruginosa CRC regulon. Sci Rep 8:16793. doi:10.1038/s41598-018-34741-9 30429516 PMC6235884

[48] Reales-Calderón JA, Corona F, Monteoliva L, Gil C, Martínez JL. 2015. Quantitative proteomics unravels that the post-transcriptional regulator CRC modulates the generation of vesicles and secreted virulence determinants of Pseudomonas aeruginosa. J Proteomics 127:352–364. doi:10.1016/j.jprot.2015.06.009 26102536

[49] McCready AR, Paczkowski JE, Cong JP, Bassler BL. 2019. An Autoinducer-independent RhlR quorum-sensing receptor enables analysis of RhlR regulation. PLoS Pathog. 15:e1007820. doi:10.1371/journal.ppat.1007820 31194839 PMC6564026

[50] Borgert SR, Henke S, Witzgall F, Schmelz S, zur Lage S, Hotop S-K, Stephen S, Lübken D, Krüger J, Gomez NO, van Ham M, Jänsch L, Kalesse M, Pich A, Brönstrup M, Häussler S, Blankenfeldt W. 2022. Moonlighting chaperone activity of the enzyme PqsE contributes to RhlR-controlled virulence of Pseudomonas aeruginosa. Nat Commun 13:1. doi:10.1038/s41467-022-35030-w 36456567 PMC9715718

[51] Simanek KA, Taylor IR, Richael EK, Lasek-Nesselquist E, Bassler BL, Paczkowski JE. 2022. The PqsE-RhlR interaction regulates RhlR DNA binding to control virulence factor production in Pseudomonas aeruginosa Microbiol Spectr 10:e0210821. doi:10.1128/spectrum.02108-21 35019777 PMC8754118

[52] Feathers JR, Richael EK, Simanek KA, Fromme JC, Paczkowski JE. 2022. Structure of the RhlR-PqsE complex from Pseudomonas aeruginosa reveals mechanistic insights into quorum-sensing gene regulation. Structure 30:1626–1636. doi:10.1016/j.str.2022.10.008 36379213 PMC9722607

[53] Malgaonkar A, Nair M. 2019. Quorum sensing in Pseudomonas aeruginosa mediated by RhlR is regulated by a small RNA PhrD. Sci Rep 9:432. doi:10.1038/s41598-018-36488-9 30674910 PMC6344545

[54] Thomason MK, Voichek M, Dar D, Addis V, Fitzgerald D, Gottesman S, Sorek R, Greenberg EP. 2019. A rhlI 5’ UTR-derived sRNA regulates RhlR-dependent quorum sensing in Pseudomonas aeruginosa. mBio 10:e02253-19. doi:10.1128/mBio.02253-19 31594819 PMC6786874

[55] Body DR. 1971. The phospholipid composition of pig lung surfactant. Lipids 6:625–629. doi:10.1007/BF02531518 4334827

[56] Gilljam H, Andersson O, Ellin A, Robertson B, Strandvik B. 1988. Composition and surface properties of the bronchial lipids in adult patients with cystic fibrosis. Clin Chim Acta 176:29–37. doi:10.1016/0009-8981(88)90171-4 3168291

[57] Wargo MJ. 2013. Homeostasis and catabolism of choline and glycine betaine: lessons from Pseudomonas aeruginosa. Appl Environ Microbiol 79:2112–2120. doi:10.1128/AEM.03565-12 23354714 PMC3623244

[58] Wang M, Schaefer AL, Dandekar AA, Greenberg EP. 2015. Quorum sensing and policing of Pseudomonas aeruginosa social cheaters. Proc Natl Acad Sci U S A 112:2187–2191. doi:10.1073/pnas.1500704112 25646454 PMC4343120

[59] Cruz RL, Asfahl KL, Van den Bossche S, Coenye T, Crabbé A, Dandekar AA. 2020. RhlR-regulated AcyL-homoserine lactone quorum sensing in a cystic fibrosis isolate of Pseudomonas aeruginosa. mBio 11:e00532-20. doi:10.1128/mBio.00532-20 32265330 PMC7157775

[60] Dandekar AA, Chugani S, Greenberg EP. 2012. Bacterial quorum sensing and metabolic incentives to cooperate. Science 338:264–266. doi:10.1126/science.1227289 23066081 PMC3587168

[61] Biswas L, Götz F. 2022. Molecular mechanisms of Staphylococcus and Pseudomonas interactions in cystic fibrosis. Front Cell Infect Microbiol 11:824042. doi:10.3389/fcimb.2021.824042 35071057 PMC8770549

[62] Hoffman LR, Richardson AR, Houston LS, Kulasekara HD, Martens-Habbena W, Klausen M, Burns JL, Stahl DA, Hassett DJ, Fang FC, Miller SI, Monack DM. 2010. Nutrient availability as a mechanism for selection of antibiotic tolerant Pseudomonas aeruginosa within the CF airway. PLoS Pathog 6:e1000712. doi:10.1371/journal.ppat.1000712 20072604 PMC2795201

[63] Maliniak ML, Stecenko AA, McCarty NA. 2016. A longitudinal analysis of chronic MRSA and Pseudomonas aeruginosa co-infection in cystic fibrosis: a single-center study. J Cyst Fibros 15:350–356. doi:10.1016/j.jcf.2015.10.014 26610860

[64] Lopez JG, Wingreen NS. 2022. Noisy metabolism can promote microbial cross-feeding. Elife 11:e70694. doi:10.7554/eLife.70694 35380535 PMC8983042

[65] Balado M, Puentes B, Couceiro L, Fuentes-Monteverde JC, Rodríguez J, Osorio CR, Jiménez C, Lemos ML. 2017. Secreted citrate serves as iron carrier for the marine pathogen Photobacterium damselae subsp damselae. Front Cell Infect Microbiol 7:361. doi:10.3389/fcimb.2017.00361 28848719 PMC5550697

[66] Frawley ER, Crouch MLV, Bingham-Ramos LK, Robbins HF, Wang W, Wright GD, Fang FC. 2013. Iron and citrate export by a major facilitator superfamily pump regulates metabolism and stress resistance in Salmonella typhimurium. Proc Natl Acad Sci U S A 110:12054–12059. doi:10.1073/pnas.1218274110 23821749 PMC3718157

[67] Michel L, Bachelard A, Reimmann C. 2007. Ferripyochelin uptake genes are involved in pyochelin-mediated signalling in Pseudomonas aeruginosa. Microbiology (Reading) 153:1508–1518. doi:10.1099/mic.0.2006/002915-0 17464065

[68] Ude J, Tripathi V, Buyck JM, Söderholm S, Cunrath O, Fanous J, Claudi B, Egli A, Schleberger C, Hiller S, Bumann D. 2021. Outer membrane permeability: antimicrobials and diverse nutrients bypass porins in Pseudomonas aeruginosa. Proc Natl Acad Sci U S A 118:e2107644118. doi:10.1073/pnas.2107644118 34326266 PMC8346889

[69] Chen R, Déziel E, Groleau MC, Schaefer AL, Greenberg EP. 2019. Social cheating in a Pseudomonas aeruginosa quorum-sensing variant. Proc Natl Acad Sci U S A 116:7021–7026. doi:10.1073/pnas.1819801116 30846553 PMC6452681

[70] García-Contreras R, Loarca D, Pérez-González C, Jiménez-Cortés JG, Gonzalez-Valdez A, Soberón-Chávez G. 2020. Rhamnolipids stabilize quorum sensing mediated cooperation in Pseudomonas aeruginosa. FEMS Microbiol Lett 367:fnaa080. doi:10.1093/femsle/fnaa080 32407463

[71] Yang Nana, Ding S, Chen F, Zhang X, Xia Y, Di H, Cao Q, Deng X, Wu M, Wong CCL, Tian X-X, Yang C-G, Zhao J, Lan L. 2015. The CRC protein participates in down-regulation of the lon gene to promote RHamnolipid production and Rhl quorum sensing in Pseudomonas aeruginosa. Mol Microbiol 96:526–547. doi:10.1111/mmi.12954 25641250

[72] Takaya A, Tabuchi F, Tsuchiya H, Isogai E, Yamamoto T. 2008. Negative regulation of quorum-sensing systems in Pseudomonas aeruginosa by ATP-dependent Lon protease. J Bacteriol 190:4181–4188. doi:10.1128/JB.01873-07 18408026 PMC2446771

[73] Yang N, Cao Q, Hu S, Xu C, Fan K, Chen F, Yang CG, Liang H, Wu M, Bae T, Lan L. 2020. Alteration of protein homeostasis mediates the interaction of Pseudomonas aeruginosa with Staphylococcus aureus. Mol Microbiol 114:423–442. doi:10.1111/mmi.14519 32323346

[74] Hall BM, Breidenstein EBM, de la Fuente-Núñez C, Reffuveille F, Mawla GD, Hancock REW, Baker TA. 2017. Two Isoforms of Clp Peptidase in Pseudomonas aeruginosa control distinct aspects of cellular physiology. J Bacteriol 199:e00568-16. doi:10.1128/JB.00568-16 27849175 PMC5237113

[75] Abdel-Haleem AM, Lewis NE, Jamshidi N, Mineta K, Gao X, Gojobori T. 2017. The emerging facets of non-cancerous warburg effect. Front Endocrinol (Lausanne) 8:279. doi:10.3389/fendo.2017.00279 29109698 PMC5660072

[76] Odoni DI, van Gaal MP, Schonewille T, Tamayo-Ramos JA, Martins Dos Santos VAP, Suarez-Diez M, Schaap PJ. 2017. Aspergillus niger secretes citrate to increase iron bioavailability. Front Microbiol 8:1424. doi:10.3389/fmicb.2017.01424 28824560 PMC5539119

[77] Li W, Lu CD. 2007. Regulation of carbon and nitrogen utilization by CbrAB and NtrBC two-component systems in Pseudomonas aeruginosa . J Bacteriol 189:5413–5420. doi:10.1128/JB.00432-07 17545289 PMC1951800

[78] Bettenworth V, van Vliet S, Turkowyd B, Bamberger A, Wendt H, McIntosh M, Steinchen W, Endesfelder U, Becker A. 2022. Frequency modulation of a bacterial quorum sensing response. Nat Commun 13:2772. doi:10.1038/s41467-022-30307-6 35589697 PMC9120067

[79] Rattray JB, Thomas SA, Wang Y, Molotkova E, Gurney J, Varga JJ, Brown SP. 2022. Bacterial quorum sensing allows graded and bimodal cellular responses to variations in population density. mBio 13:e0074522. doi:10.1128/mbio.00745-22 35583321 PMC9239169

[80] Mukherjee S, Bassler BL. 2019. Bacterial Quorum sensing in complex and dynamically changing environments. Nat Rev Microbiol 17:371–382. doi:10.1038/s41579-019-0186-5 30944413 PMC6615036

[81] McGranahan N, Swanton C. 2015. Biological and therapeutic impact of intratumor heterogeneity in cancer evolution. Cancer Cell 27:15–26. doi:10.1016/j.ccell.2014.12.001 25584892

[82] Marusyk A, Janiszewska M, Polyak K. 2020. Intratumor heterogeneity: the rosetta stone of therapy resistance. Cancer Cell 37:471–484. doi:10.1016/j.ccell.2020.03.007 32289271 PMC7181408

[83] Bozic I, Nowak MA. 2014. Timing and heterogeneity of mutations associated with drug resistance in metastatic cancers. Proc Natl Acad Sci U S A 111:15964–15968. doi:10.1073/pnas.1412075111 25349424 PMC4234551

[84] Demers EG, Biermann AR, Masonjones S, Crocker AW, Ashare A, Stajich JE, Hogan DA. 2018. Evolution of drug resistance in an antifungal-naive chronic Candida lusitaniae infection. Proc Natl Acad Sci U S A 115:12040–12045. doi:10.1073/pnas.1807698115 30389707 PMC6255150

[85] Shanks RMQ, Caiazza NC, Hinsa SM, Toutain CM, O’Toole GA. 2006. Saccharomyces cerevisiae-based molecular tool kit for manipulation of genes from gram-negative bacteria. Appl Environ Microbiol 72:5027–5036. doi:10.1128/AEM.00682-06 16820502 PMC1489352

[86] Kasetty S, Mould DL, Hogan DA, Nadell CD. 2021. Both Pseudomonas aeruginosa and Candida albicans accumulate greater biomass in dual-species biofilms under flow. mSphere 6:e0041621. doi:10.1128/mSphere.00416-21 34160236 PMC8265656

[87] Tang Y, Horikoshi M, Li W. 2001. Ggfortify: unified interface to visualize statistical results of popular R packages. The R Journal 8:474. doi:10.32614/RJ-2016-060

[88] Wickham H. 2016. Ggplot2: elegent graphics for data analysis. Springer-Verlag New York, Cham.

[89] Dawson C. 2022. Ggprism: a ’ggplot2’ extension inspired by ’graphpad prism

[90] Miller JH. 1992. A short course in bacterial genetics: a laboratory manual and handbook for Escherichia coli and related bacteria. Cold Spring Harbor Press.

[91] Hammond JH, Dolben EF, Smith TJ, Bhuju S, Hogan DA. 2015. Links between Anr and quorum sensing in Pseudomonas aeruginosa biofilms. J Bacteriol 197:2810–2820. doi:10.1128/JB.00182-15 26078448 PMC4524035

