# Supplementary material for: Citrate cross-feeding by Pseudomonas aeruginosa supports lasR mutant fitness
Source: mBio. 2024 Jan 23;15(2):e01278-23. doi: 10.1128/mbio.01278-23 (PMC10865840; doi:10.1128/mbio.01278-23)
Supplement: Supplemental figures — Figures S1-S5. [file mbio.01278-23-s0001.pdf]

Mould 2023 mBio Supplement

Citrate cross-feeding by *Pseudomonas aeruginosa* supports *lasR* mutant fitness

Dallas L. Mould,[a] Carson E. Finger,[a] Amy Conaway,[a] Nico Botelho,[a] Stacie E. Stuut,[a] Deborah A. Hogan[a,*]
Geisel School of Medicine at Dartmouth, Department of Microbiology and Immunology, Hanover, NH USA[a];

Address correspondence to Deborah A. Hogan, dhogan@dartmouth.edu.

SUPPLEMENTAL MATERIALS

The following supplemental materials are associated with the manuscript:

Table S1 Relative intracellular metabolites detected for LasR+ and LasR- paired isolates on LB or Artificial Sputum Medium (ASM).[a]

[a]See attached excel file with raw and normalized metabolite counts with differential abundance and significance indicated.

Table S2 Strains and plasmids used in this study[b]

[b]See attached PDF file with strain identifier, description and source listed for each strain and plasmid.

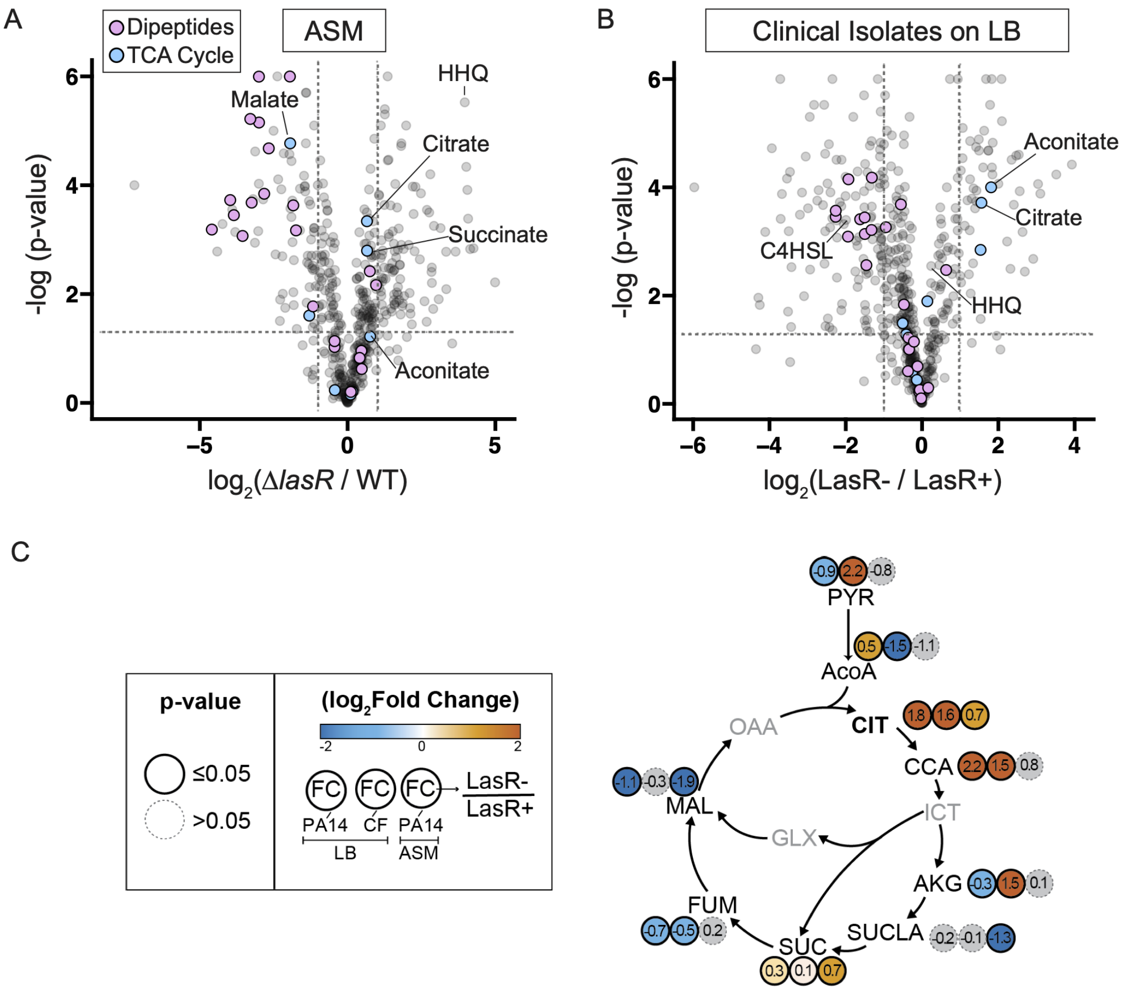

Fig. S1 Citrate is the only tricarboxylic acid (TCA) cycle intermediate significantly enriched in LasR- cells across genetic background and media type. Volcano plots showing differential intracellular metabolite counts ($\log_2$ LasR-/LasR+) for the laboratory isolates PA14 and Δ*lasR* on Artificial Sputum Medium (ASM) (A) or the LasR- clinical isolate compared to its closely related LasR+ progenitor on LB (B) relative to the $-\log_{10}$ (P value). Metabolites are colored based on their categorization as dipeptides (lavender) or inclusion in the TCA cycle (light blue). 4-hydroxy-2-heptylquinoline (HHQ) and N-butanoyl-L-homoserine lactone (C4HSL) which are known to be enriched and depleted in *lasR* mutants, respectively are also indicated. C4HSL was not significantly different in the ASM dataset between strains. C. The $\log_2$ fold change and statistical significance of metabolites noted for LasR- over LasR+ strains in all three conditions tested (3 circles, left to right): PA14 Δ*lasR* / WT on LB, LasR- DH2415/ LasR+ DH2417 on LB, and Δ*lasR* / WT on artificial sputum medium (ASM). Circle fill is colored by relative $\log_2$ fold change as indicated by scale ranging from -2 to 2. Significance as determined by Welch's t-test is indicated by circle outline (P value ≤ 0.05, solid outline and P value > 0.05 grey, dotted). Metabolites listed in grey text without numbers were not detected in our analysis.

Mould 2023 mBio Supplement Mould 2023 mBio Supplement Mould 2023 mBio Supplement Mould 2023 mBio Supplement Mould 2023 mBio Supplement Mould 2023 mBio Supplement

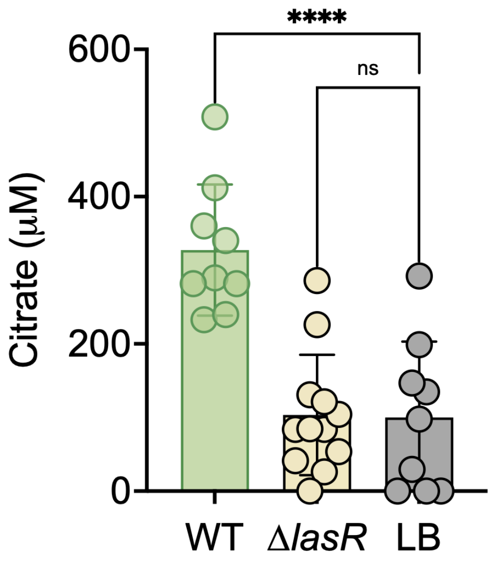

Fig. S2 Total extracellular citrate in cell-free supernatant taken from 5 mL LB cultures of PA14 wild type or the Δ*lasR* strain compared to the uninoculated LB medium blank. Statistical significance as determined by One-Way ANOVA with Dunnett's multiple comparison test, ****, P value < 0.0001 and ns, not significant (P value = 0.9902 for the LB and Δ*lasR* comparison).

Mould 2023 mBio Supplement Mould 2023 mBio Supplement Mould 2023 mBio Supplement Mould 2023 mBio Supplement Mould 2023 mBio Supplement Mould 2023 mBio Supplement

Mould 2023 mBio Supplement

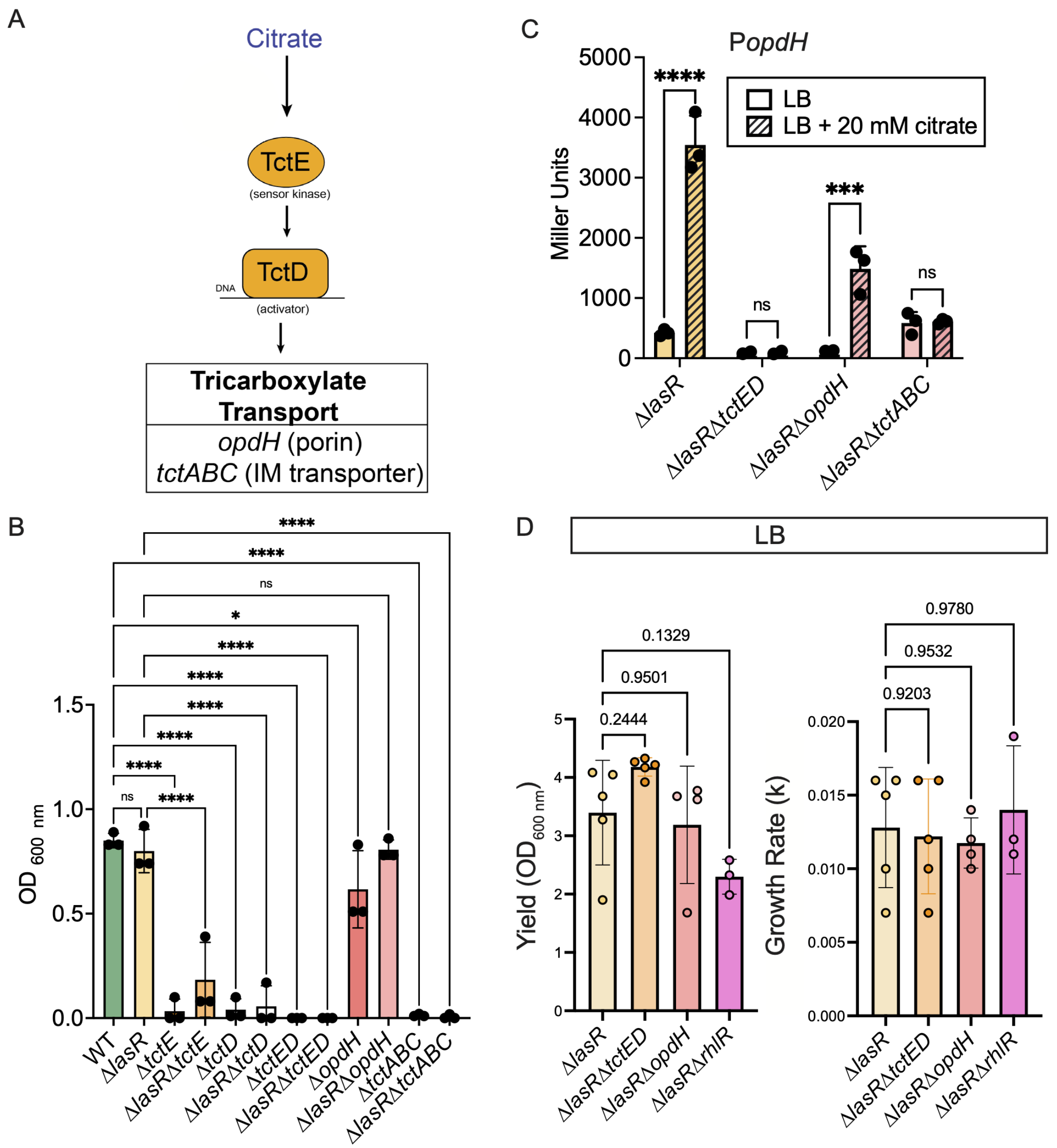

Fig. S3 TctED positively regulate citrate transport necessary for growth on citrate but not LB. A. Model illustrating the positive control both the sensor kinase TctE and response regulator TctD have on select genes involved in tricarboxylate transport (i.e. *opdH* and *tctABC*) in response to citrate. OpdH is a porin located in the outer membrane and TctABC is an inner membrane (IM) transporter. B. Growth in M63 base containing 10 mM citrate as a sole carbon source after 24 h from a single colony inoculum for the indicated strains. C. Quantification of P*opdH* – GFP – *lacZ* via beta-galactosidase assay for the indicated strains when grown for 24 h as colony biofilms on LB agar or LB agar supplemented with 20 mM citrate. D. Growth yield after 8 h subculture and exponential growth rate in 5 mL LB cultures.

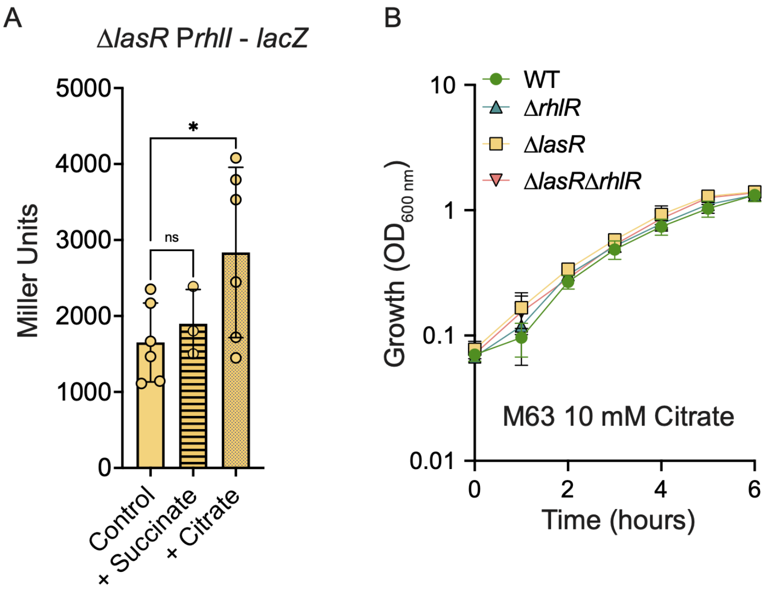

Fig. S4 RhlR is activated by citrate and not succinate, but *rhlR* does not impact growth on citrate. A. Quantification of P*rhlI* – *lacZ* via beta-galactosidase assay for the Δ*lasR* strain grown on LB agar (control) or LB agar supplemented with either 20 mM succinate (+ Succinate) or 20 mM citrate (+ Citrate). Statistical significance as determined by mixed-effects analysis with Dunnett's multiple comparison test relative to "Control": *, P value < 0.05. Succinate comparison was not significant. B. Growth curves of indicated strains in M63 medium with 10 mM citrate as a sole carbon source.

A

Sample	Count	MFI
Δ*lasR* monoculture GFP	99487	1100
Δ*lasR* co-culture GFP	29525	3149

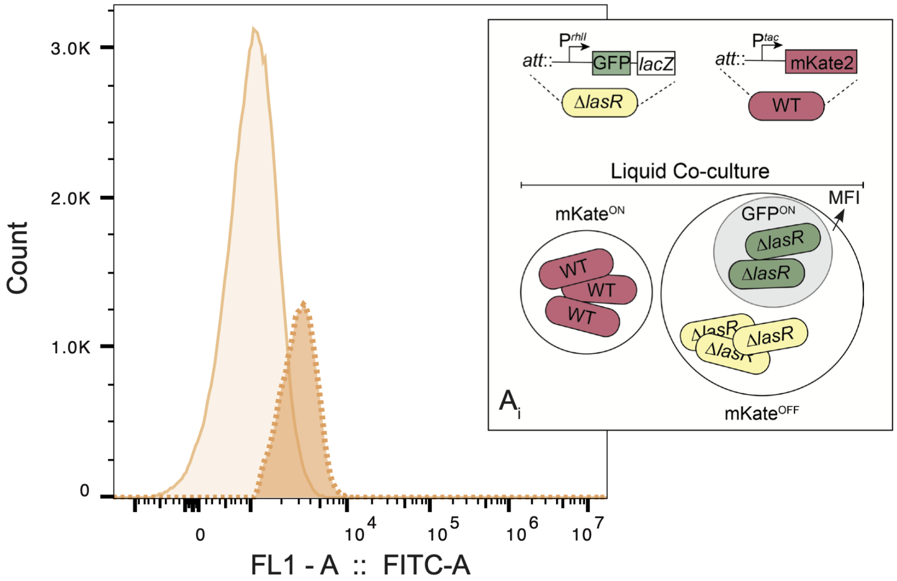

Fig. S5 In co-culture with wild type, Δ*lasR* cells exhibit increased RhlR-dependent *rhlI* promoter activity at the cellular level. A. Representative histogram of mKate2 negative counts by expression of green fluorescent protein (GFP), which was detected by FITC channel, of the Δ*lasR* P*rhlI* – GFP – *lacZ* cells in monoculture (light beige, solid line) or co-culture (darker beige, dotted line) with a wildtype strain expressing mKate2 constitutively under the P*tac* promoter at a neutral site (illustrated in inset). Cell counts and median fluorescence intensity (MFI) indicated for cells lacking mKate expression (mKate^{OFF}) that exhibit GFP fluorescence (GFP^{ON}) after 6h subculture in LB.

Mould 2023 mBio Supplement Mould 2023 mBio Supplement Mould 2023 mBio Supplement Mould 2023 mBio Supplement Mould 2023 mBio Supplement Mould 2023 mBio Supplement
