# Supplementary material for: Citrate cross-feeding by Pseudomonas aeruginosa supports lasR mutant fitness
Source: mBio. 2024 Jan 23;15(2):e01278-23. doi: 10.1128/mbio.01278-23 (PMC10865840; doi:10.1128/mbio.01278-23)
Supplement: Table S2 — Strains and plasmids. [file mbio.01278-23-s0003.pdf]

Supplemental Table 2

Table S2. Strains and plasmids used in this study

Strain	*Strain.ID*	*Description*	*Source*
P. aeruginosa			
PA14 WT	DH122	Laboratory reference strain	(1)
PA14 Δ*lasR*	DH164	DH122 with in-frame deletion of *lasR* (*PA14_45960*)	(2)
PA14 Δ*phz*	DH933	In-frame deletions of *phzA1-G1* and *phzA2-G2*	(3)
PA14 Δ*rhlR*	DH2742	PA14 WT (DH122) with in-frame deletion of *rhlR*	(4)
PA14 Δ*lasR*Δ*rhlR*	DH2944	In-frame deletion of *lasR* and *rhlR*	(4)
NC-AMT0101-1-2	DH2417	Chronic CF lung infection isolate with functional LasR allele, parent of NC-AMT0101-1	(5)
NC-AMT0101-1-1	DH2415	Chronic CF lung infection isolate related to DH2417 with LasR LOF (frameshift) allele	(5)
PA14 Δ*tctE*	DH4175	PA14 WT (DH122) with in-frame deletion of *tctE*	This study
PA14 Δ*tctD*	DH4176	PA14 WT (DH122) with in-frame deletion of *tctD*	This study
PA14 Δ*tctED*	DH4177	PA14 WT (DH122) with in-frame deletion of *tctED*	This study
PA14 Δ*tctABC*	DH4178	PA14 WT (DH122) with in-frame deletion of *tctABC*	This study
PA14 Δ*lasR*Δ*tctD*	DH4179	PA14 Δ*lasR* (DH164) with in frame deletion of *tctD*	This study

PA14 Δ*lasR*Δ*tctE*	DH4180	PA14 Δ*lasR* (DH164) with in-frame deletion of *tctE*	This study
PA14 Δ*lasR*Δ*tctED*	DH4181	PA14 Δ*lasR* (DH164) with in frame deletion of *tctED*	This study
PA14 Δ*lasR*Δ*tctABC*	DH4182	PA14 Δ*lasR* (DH164) with in-frame deletion of *tctABC*	This study
PA14 Δ*opdH*	DH4183	PA14 WT (DH122) with in-frame deletion of *opdH*	This study
PA14 Δ*lasR*Δ*opdH*	DH4184	PA14 Δ*lasR* (DH164) with in-frame deletion of *opdH*	This study
PA14 Δ*lasR*Δ*opdH* + *opdH*	DH4185	PA14 Δ*lasR*Δ*opdH* (DH4184) with complementation of *opdH* at the native locus	This study
PA14 Δ*cbrB*	DH3920	PA14 WT (DH122) with in-frame deletion of *cbrB* (PA14_62540)	(6)
PA14 Δ*lasR*Δ*cbrB*	DH3924	PA14 Δ*lasR* (DH164) with in-frame deletion of *cbrB* (PA14_62540)	(6)
PA14 Δ*lasR*Δ*cbrB* + *cbrB*	DH3925	PA14 Δ*lasR*Δ*cbrB* (DH3924) with complementation of *cbrB* (PA14_62540) at the native locus	(6)
PA14 Δ*lasR*Δ*cbrB*Δ*crc*	DH3926	PA14 Δ*lasR*Δ*cbrB* (DH3924) with in-frame deletion of *crc* (PA14_70390)	(6)
PA14 Δ*lasR*Δ*cbrB*Δ*crc* + *crc*	DH4186	PA14 Δ*lasR*Δ*cbrB*Δ*crc* (DH3926) with complementation of *crc* (PA14_70390) at the native locus	This study
PA14 WT *att::lacZ*	DH22	PA14 WT with constitutive expression of *lacZ*	Roberto Kolter (7, 8)
PA14 Δ*lasR*Δ*betAB*	DH4187	In-frame deletion of *lasR* in Δ*betAB* (DH1316)	This study

PA14 Δ*lasR*Δ*betAB*Δ*tctED*	DH4188	In-frame deletion of *tctED* in Δ*lasR*Δ*betAB* (DH4187)	This study
PA14 Δ*lasR* P*rhlI*-GFP-*lacZ*	DH3313	PA14 Δ*lasR* (DH164) expressing P*rhlI*-GFP-*lacZ* promoter fusion at the *att*::Tn7 site	(9)
PA14 Δ*lasR*Δ*rhlR* P*rhlI*-GFP-*lacZ*	DH3309	PA14 Δ*lasR*Δ*rhlR* (DH2944) expressing P*rhlI*-*lacZ* promoter fusion at the *att*::Tn7 site	(4)
PA14 Δ*lasR*Δ*cbrB* P*rhlI*-GFP- *lacZ*	DH4189	PA14 Δ*lasR*Δ*cbrB* (DH3924) expressing P*rhlI*-GFP-*lacZ* promoter fusion at the att::Tn7 site	This study
PA14 Δ*lasR*Δ*tctED* P*rhlI*-GFP-*lacZ*	DH4190	PA14 Δ*lasR*Δ*tctED* (DH4181) expressing P*rhlI*-*lacZ* promoter fusion at the att::Tn7 site	This study
PA14 Δ*lasR*Δ*opdH* P*rhlI*-GFP-*lacZ*	DH4191	PA14 Δ*lasR*Δ*opdH* (DH4184) expressing P*rhlI*-*lacZ* promoter fusion at the *att*::Tn7 site	This study
PA14 Δ*lasR*Δ*rhlI* P*rhlI*-GFP-*lacZ*	DH4192	PA14 Δ*lasR*Δ*rhlI* (DH238) expressing P*rhlI*-GFP-*lacZ* promoter fusion at the att::Tn7 site	This study
PA14 WT P*opdH*-GFP-*lacZ*	DH4193	PA14 WT (DH122) expressing P*opdH*-GFP-*lacZ* promoter fusion at the att::Tn7 site	This study
PA14 Δ*lasR* P*opdH*-GFP-*lacZ*	DH4194	PA14 Δ*lasR* (DH164) expressing P*opdH*-GFP-l*acZ* promoter fusion at the att::Tn7 site	This study
PA14 Δ*lasR*Δ*tctD* P*opdH*-GFP-*lacZ*	DH4195	PA14 Δ*lasR*Δ*tctD* (DH4179) expressing P*opdH*-GFP-*lacZ* promoter fusion at the att::Tn7 site	This study
PA14 Δ*lasR*Δ*tctE* P*opdH*-GFP-*lacZ*	DH4196	PA14 Δ*lasR*Δ*tctE* (DH4180) expressing P*opdH*-GFP-*lacZ*	This study

		promoter fusion at the att::Tn7 site	
PA14 Δ*lasR*Δ*tctED* P*opdH*-GFP-*lacZ*	DH4197	PA14 Δ*lasR*Δ*tctED* (DH4181) expressing P*opdH*-GFP-*lacZ* promoter fusion at the att::Tn7 site	This study
PA14 Δ*lasR*Δ*tctABC* P*opdH*-GFP-*lacZ*	DH4198	PA14 Δ*lasR*Δ*tctABC* (DH4182) expressing P*opdH*-GFP-*lacZ* promoter fusion at the att::Tn7 site	This study
PA14 Δ*lasR*Δ*opdH* P*opdH*-GFP-*lacZ*	DH4199	PA14 Δ*lasR*Δ*opdH* (DH4184) expressing P*opdH*-GFP-*lacZ* promoter fusion at the att::Tn7 site	This study
PA14 att::P*tac*-mKate	DH4200	PA14 WT with two tandem copies of mKate2 under a synthetic *tac* promoter integrated at the Tn7 *att* site	This study
E. coli			
S17 λpir	DH71	Used as a conjugation partner for introducing pMQ30 and GH121-based plasmids.	
DH5a	DH51	Used to store/replicate plasmids.	Invitrogen
Plasmids			
pMQ30 EV	DH962	Allelic replacement vector for use in yeast cloning, Gm^R	(10)
GH121 EV	DH2830	For inserting sequences at the *att*::Tn7 site via allelic replacement; Gm^R	(11)
GH121_Ptac_mKate	DH3688	Two tandem copies of *mKate2* (codon optimized; each with its own ribosome binding site and different codon composition to prevent excision by recombination) under a synthetic tac promoter, for	This study;(12)

		integration at the *att:*Tn7 site; Gm^R	
GH121_P*rhlI-lacZ*	DH3314	GFP-*lacZ* under control of the *rhlI* promoter, for integration at the *att*::Tn7 site; Gm^R	(4)
GH121_P*opdH-lacZ*	DH4201	GFP-*lacZ* under control of the *opdH* promoter (position -210 to 3), for integration at the *att*::Tn7 site; Gm^R	This study
p*lasR*_KO	DH133	PA14 *lasR* in-frame deletion construct; Gm^R	(2)
pMQ30_*crc*_KON	DH3511	*crc* (PA14_70390) native locus complementation construct; Gm^R	(6)
pEX18_*tctED*_KO	DH4202	PA14 *tctED* in-frame deletion construct; Gm^R	(13)
pMQ30_*opdH*_KO	DH4203	*opdH* in-frame deletion construct; Gm^R	This study
pMQ30_*opdH*_KON	DH4204	*opdH* complementation construct at native locus; Gm^R	This study
pMQ30_*tctD*_KO	DH4205	*tctD* in-frame deletion construct; Gm^R	This study
pMQ30_*tctE*_KO	DH4206	*tctE* in-frame deletion construct; Gm^R	This study
pMQ30_*tctABC*_KO	DH4207	*tctABC* in-frame deletion construct; Gm^R	This study

1. Rahme LG, Stevens EJ, Wolfort SF, Shao J, Tompkins RG, Ausubel FM. 1995. Common virulence factors for bacterial pathogenicity in plants and animals. Science 268:1899-902.
2. Hogan DA, Vik A, Kolter R. 2004. A *Pseudomonas aeruginosa* quorum-sensing molecule influences *Candida albicans* morphology. Mol Microbiol 54:1212-23.

3. Dietrich LE, Price-Whelan A, Petersen A, Whiteley M, Newman DK. 2006. The phenazine pyocyanin is a terminal signalling factor in the quorum sensing network of *Pseudomonas aeruginosa*. Mol Microbiol 61:1308-21.
4. Harty CE, Martins D, Doing G, Mould DL, Clay ME, Occhipinti P, Nguyen D, Hogan DA. 2019. Ethanol stimulates trehalose production through a SpoT-DksA-AlgU dependent pathway in *Pseudomonas aeruginosa*. Journal of Bacteriology doi:10.1128/jb.00794-18:JB.00794-18.
5. Smith EE, Buckley DG, Wu Z, Saenphimmachak C, Hoffman LR, D'Argenio DA, Miller SI, Ramsey BW, Speert DP, Moskowitz SM, Burns JL, Kaul R, Olson MV. 2006. Genetic adaptation by *Pseudomonas aeruginosa* to the airways of cystic fibrosis patients. Proc Natl Acad Sci U S A 103:8487-92.
6. Mould DL, Stevanovic M, Ashare A, Schultz D, Hogan DA. 2022. Metabolic basis for the evolution of a common pathogenic *Pseudomonas aeruginosa* variant. Elife 11.
7. Wang Z, Xiong G, Lutz F. 1995. Site-specific integration of the phage phi CTX genome into the *Pseudomonas aeruginosa* chromosome: characterization of the functional integrase gene located close to and upstream of *attP*. Mol Gen Genet 246:72-9.
8. Choi KH, Schweizer HP. 2006. mini-Tn7 insertion in bacteria with single *att*Tn7 sites: example *Pseudomonas aeruginosa*. Nat Protoc 1:153-61.
9. Mould DL, Botelho NJ, Hogan DA. 2020. Intraspecies signaling between common variants of *Pseudomonas aeruginosa* increases production of quorum-sensing-controlled virulence factors. mBio 11.
10. Shanks RM, Caiazza NC, Hinsa SM, Toutain CM, O'Toole GA. 2006. *Saccharomyces cerevisiae*-based molecular tool kit for manipulation of genes from gram-negative bacteria. Appl Environ Microbiol 72:5027-36.
11. Heussler GE, Cady KC, Koeppen K, Bhuju S, Stanton BA, O’Toole GA. 2015. Clustered Regularly Interspaced Short Palindromic Repeat-Dependent, Biofilm-Specific Death of *Pseudomonas aeruginosa* Mediated by Increased Expression of Phage-Related Genes. mBio 6:e00129-15.
12. Kasetty S, Mould DL, Hogan DA, Nadell CD. 2021. Both *Pseudomonas aeruginosa* and *Candida albicans* accumulate greater biomass in dual-species biofilms under flow. mSphere 6:e0041621.
13. Zhang L, Fritsch M, Hammond L, Landreville R, Slatculescu C, Colavita A, Mah TF. 2013. Identification of genes involved in *Pseudomonas aeruginosa* biofilm-specific resistance to antibiotics. PLoS One 8:e61625.
